# Internally tagged Vps10p-domain receptors reveal uptake of the neurotrophin BDNF

**DOI:** 10.1016/j.jbc.2023.105216

**Published:** 2023-09-01

**Authors:** Marcel Klein, Antonio Virgilio Failla, Guido Hermey

**Affiliations:** 1 Institute for Molecular and Cellular Cognition, Center for Molecular Neurobiology Hamburg, University Medical Center Hamburg-Eppendorf, Hamburg, Germany; 2 Microscopy Imaging Facility, University Medical Center Hamburg-Eppendorf, Hamburg, Germany

**Keywords:** sortilin, SORLA, SorCS, neurotrophin, BDNF, protein sorting, receptor internalization, live-cell imaging

## Abstract

The Vps10p-domain (Vps10p-D) receptor family consists of Sortilin, SorLA, SorCS1, SorCS2, and SorCS3. They mediate internalization and intracellular sorting of specific cargo in various cell types, but underlying molecular determinants are incompletely understood. Deciphering the dynamic intracellular itineraries of Vps10p-D receptors is crucial for understanding their role in physiological and cytopathological processes. However, studying their spatial and temporal dynamics by live imaging has been challenging so far, as terminal tagging with fluorophores presumably impedes several of their protein interactions and thus functions. Here, we addressed the lack of appropriate tools and developed functional versions of all family members internally tagged in their ectodomains. We predict folding of the newly designed receptors by bioinformatics and show their exit from the endoplasmic reticulum. We examined their subcellular localization in immortalized cells and primary cultured neurons by immunocytochemistry and live imaging. This was, as far as known, identical to that of wt counterparts. We observed homodimerization of fluorophore-tagged SorCS2 by coimmunoprecipitation and fluorescence lifetime imaging, suggesting functional leucine-rich domains. Through ligand uptake experiments, live imaging and fluorescence lifetime imaging, we show for the first time that all Vps10p-D receptors interact with the neurotrophin brain-derived neurotrophic factor and mediate its uptake, indicating functionality of the Vps10p-Ds. In summary, we developed versions of all Vps10p-D receptors, with internal fluorophore tags that preserve several functions of the cytoplasmic and extracellular domains. These newly developed fluorophore-tagged receptors are likely to serve as powerful functional tools for accurate live studies of the individual cellular functions of Vps10p-D receptors.

The Vps10p-domain (Vps10p-D) receptor family consists of the five type I transmembrane proteins, Sortilin, SorLA, SorCS1, SorCS2, and SorCS3, in mammals (1). All family members are predominantly expressed in the developing and adult nervous system with specific spatiotemporal expression patterns (2, 3, 4, 5, 6). All, with exception of SorCS3, are also expressed in varying peripheral tissues (7, 8, 9, 10, 11). A large number of genetic linkage analysis and functional studies demonstrated an association of the receptors with several neurodegenerative and psychiatric disorders, including Alzheimer’s disease and autism spectrum disorders (12, 13, 14, 15, 16, 17, 18, 19), and metabolic diseases, including diabetes, atherosclerosis, and hypercholesterolemia (20, 21, 22, 23, 24).

The shared hallmark of the family is the N-terminal Vps10p-D, which was first described in the yeast sorting receptor Vps10p (25). Because of the intracellular sorting function of yeast Vps10p, sorting properties were also proposed for the mammalian Vps10p-D receptors. In agreement, these bind different ligands and mediate their secretion, internalization, and sorting through the endosomal system (1, 26, 27). All family members present a large luminal/extracellular moiety followed by a transmembrane domain and a short cytoplasmic tail (Fig. 1*A*). The Vps10p-D forms at its N terminus a β-propeller resembling a conical funnel with binding sites for different ligands followed by two small cysteine-rich domains, named 10CC module (28, 29, 30). In Sortilin, the luminal/extracellular moiety comprises only the Vps10p-D (8, 31). In the SorCS subgroup (SorCS1–3), a leucine-rich domain is located between the Vps10p-D and the transmembrane domain. It contains not only imperfect leucine-rich repeats but also regions with homology to polycystic kidney disease domains and is thought to allow receptor dimerization (10, 30, 32). In SorLA, the Vps10p-D is followed by structural motifs shared with the low-density lipoprotein receptor family, a YWTD β-propeller domain, an epidermal growth factor precursor type repeat, low-density lipoprotein receptor class A repeats, and fibronectin type III repeats (7).

**Figure 1:**
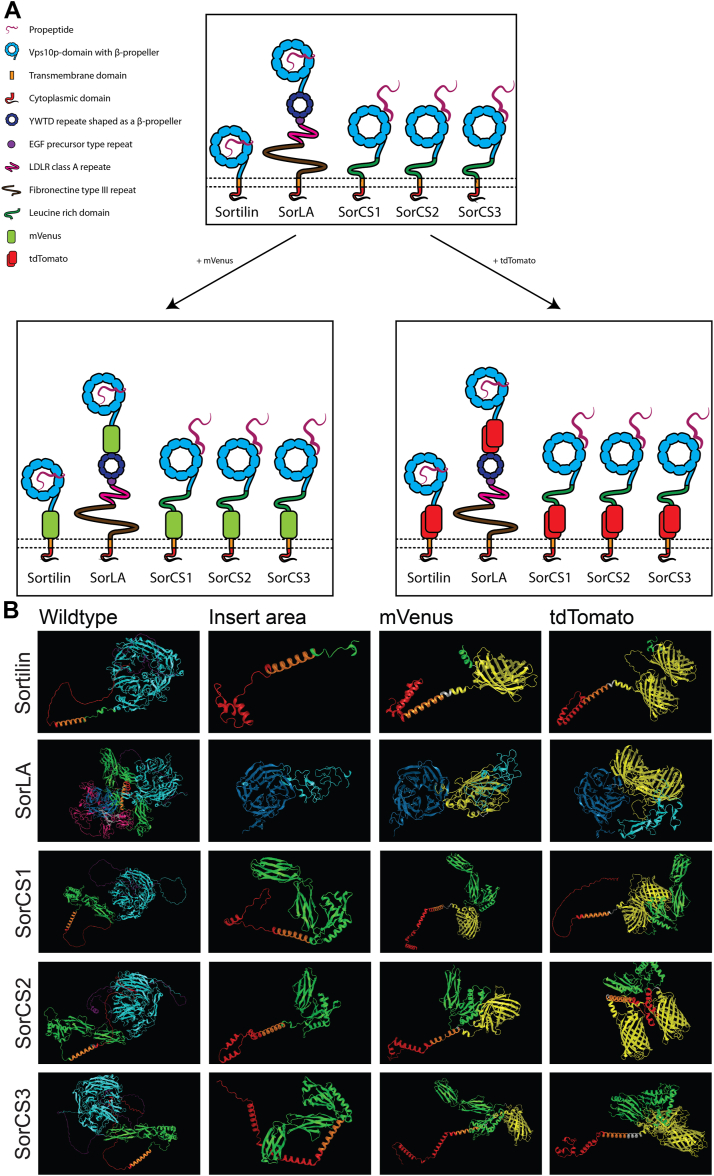
**Domain organization and structural analysis of the Vps10p-D receptors with and without fluorophore insertion.***A*, domain organization of the Vps10p-D receptors (*top*) and design of fluorophore-tagged versions of the Vps10p-D receptors, tagged with mVenus (*bottom left*) and tagged with tdTomato (*bottom right*). *B*, structural predictions of the wt Vps10p-D receptors and their domains surrounding the insert area before and after mVenus or tdTomato insertion using AlphaFold 2.0. *Purple* represents the propeptide, *light blue* the Vps10p domain, *dark blue* the YWTD repeat, *gray* the EGF precursor type repeat, *magenta* the LDLR class A repeat, *green* the fibronectin type III repeat in SorLA and the leucine-rich domain in the SorCS subgroup, *orange* the transmembrane domain, *red* the cytoplasmic domain, and *yellow* the fluorophores. EGF, epidermal growth factor; LDLR, low-density lipoprotein receptor; Vps10p-D, Vps10p-domain.

An N-terminal propeptide precedes all Vps10p-Ds (Fig. 1*A*), which in Sortilin and SorLA, but not in the SorCS subgroup, is binding to their own Vps10p-Ds, causing an inability of additional binding of most ligands to this domain (33, 34, 35, 36, 37). The proprotein convertase furin cleaves the propeptides of all family members within the trans-Golgi network (TGN). This proteolytic step activates the Vps10p-D of Sortilin and SorLA for ligand binding. At least in SorCS1, alternative functional furin cleavage sites for the propetide exist, indicating possible differential proprotein convertase processing (38).

The receptors interact with a broad range of transmembrane and secreted proteins, including trophic factors. In particular, the roles of Sortilin and SorCS2 in neuronal plasticity and apoptotic signaling by interacting through their Vps10p-Ds with neurotrophins have been studied. The neurotrophins nerve growth factor (NGF) and brain-derived neurotrophic factor (BDNF) are synthesized as precursors, so called proneurotrophins (39). Proteolytic cleavage of the prodomain releases the mature neurotrophin. Both the pro and mature forms can be secreted and display distinct and often opposing biological activities. Mature neurotrophins bind their cognate tropomyosin receptor kinase (Trk) family receptors promoting growth and survival of neurons, whereas proneurotrophins form a ternary complex with p75 neurotrophin receptor (p75NTR) and Sortilin or SorCS2 to induce apoptotic signaling. Both, pro and mature neurotrophins bind to Trks, p75NTR, and the two Vps10p-D receptors. In general, mature neurotrophins bind p75NTR and Trks with higher affinity, whereas the affinity of proneurotrophins seems to be higher for the two Vps10p-D receptors (30, 34, 40, 41, 42). The latter assumption is mainly based on binding studies of pro-BDNF, pro-NGF, and NGF to Sortilin and SorCS2. However, there are exceptions as SorCS3 binds mature NGF with higher affinity than pro-NGF (43). Because of the homologous structure of the Vps10p-Ds within the receptor family, it has been hypothesized that all family members bind neurotrophins, but so far, this concept awaits experimental corroboration.

The cytoplasmic domains of all Vps10p-D receptors contain internalization motifs and varying adaptor protein-binding sites. SorCS1 is expressed as different splice variants with alternative cytoplasmic domains (33). Notably, the variant SorCS1b is the only Vps10p-D receptor lacking a functional internalization motif in its cytoplasmic domain and is localized predominantly to the cell surface (33). In contrast, all other SorCS1 splice variants, such as the here studied SorCS1c-α, and all other Vps10p-D receptors are endocytic receptors targeted mainly to Golgi and endosomal compartments (1, 26, 27).

The assumption that Vps10p-D receptors convey intracellular sorting is based on the interaction of their cytoplasmic domains with various adaptor proteins involved in endosomal targeting (44, 45, 46, 47, 48, 49, 50, 51). Moreover, studies employing knockout models revealed altered localization or mistargeting of interacting proteins (24, 49, 52, 53, 54, 55, 56, 57), and the expression of chimeric receptor constructs demonstrated internalization and intracellular targeting of ligands or rescued specific phenotypes in knockout models (6, 33, 43, 46, 47, 48). In previous studies, the subcellular localization of endogenous Vps10p-D receptors was determined by subcellular fractionation or by immunocytochemistry of fixed cells. There have been also attempts to perform direct and live imaging of Vps10p-D receptor localization and trafficking.

The most widely applied strategy for live imaging of proteins is the use of fluorescent protein tags. Such fusion proteins enable the noninvasive analysis of protein localization and dynamics in living cells owing to the unique ability of GFP-like fluorescent proteins to form chromophores autocatalytically without the involvement of external enzymes and cofactors (58). When genetically fused to a protein of interest, fluorophores offer an exquisite labeling specificity. In previous studies, fluorophores were fused N-terminal to the transmembrane and cytoplasmic domains of Sortilin or SorLA (59, 60). These constructs lack the receptor-specific luminal/extracellular moieties but revealed important information on the subcellular targeting and transport dynamics mediated by the respective cytoplasmic domains.

In a number of other studies, full-length Sortilin, SorLA, or SorCS2 was tagged at the C terminus with a fluorophore (45, 49, 55, 61, 62, 63). These constructs retained full functionality of the respective luminal/extracellular domains, but the C-terminal tag likely interferes with different interactions of the cytosolic tails. Binding of several cytosolic adaptor proteins, such as GGAs and PDZ-domain proteins, to their specific amino acid motifs depends on a free C terminus (64, 65). Notably, already small C-terminal peptide tags, such as a myc tag, impede GGA interaction (64). GGAs are known functional Sortilin and SorLA interactors (48, 49, 64, 66, 67), the PDZ-domain protein PICK1 targets SorLA and SorCS3 (44, 50) and additional yet unidentified C-terminal interactors are most likely. Therefore, C-terminal tagging probably leads to aberrant subcellular targeting of Vps10p-D receptors. Hence, other tagging strategies are needed to study Vps10p-D receptors by live and *in vivo* imaging. We hypothesized that internal fluorophore tagging in the luminal/extracellular moiety between structural modules results in functional tools to study Vps10p-D receptors in live experiments. We excluded an N-terminal tag because it would be cleaved off by furin in the secretory pathway, furin cleavage is critical for some receptor function, and a fluorophore tag following the furin cleavage site probably interferes with the ligand binding capacity of the Vps10p-D. We also excluded a C-terminal tag because it impedes specific interactions with adaptor proteins binding to C-terminal sites and an insertion of a fluorophore tag between the transmembrane and the cytoplasmic domains because several sorting motifs depend on the correct spacing relative to the plasma membrane (68). Therefore, we designed genetically encoded versions of the receptors harboring an internal tag in the luminal/extracellular moiety between functional modules and the transmembrane domain.

Previously, we presented versions of different SorCS1 splice variants with an internal mVenus tag between their transmembrane and extracellular domains to analyze their intracellular transport and differences in amyloid precursor protein sorting (69). Here, we applied this tagging strategy to all members of the Vps10p-D receptor family. We validate the structure of the internally tagged constructs by bioinformatics and their expression and subcellular localization in fixed cells and by live imaging. We assess dimerization of constructs of the SorCS subgroup by coimmunoprecipitation (co-IP) and fluorescence lifetime imaging microscopy (FLIM). We demonstrate that all internally tagged Vps10p-D receptors interact with BDNF and facilitate its cellular uptake by imaging-based internalization experiments and FLIM. These findings support the utility of the newly designed fluorophore-tagged receptors and indicate that they are valuable tools for studying intracellular sorting and transport as well as receptor–ligand interaction.

## Results

### Design and structure prediction of fluorophore-tagged Vps10p-D receptors

**Fig S1:**
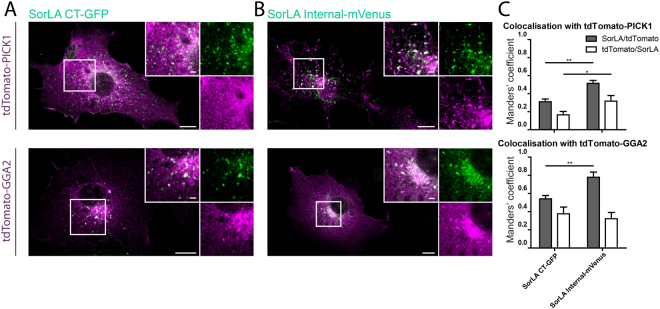
**C-terminal tagging of SorLA reduces colocalization with PICK1 and GGA2.***A*, colocalization analysis of C-terminally GFP tagged SorLA (SorLA CT-GFP). *B*, colocalization analysis of internally mVenus-tagged SorLA (SorLA Internal-mVenus) with tdTomato-PICK1 or tdTomato-GGA2. Confocal images of HeLa cells transfected with SorLA CT-GFP (*green*, *left*) or SorLA Internal-mVenus (*green*, *right*) and tdTomato-PICK1 (*red*, *top*) or tdTomato-GGA2 (*red*, *bottom*). Fluorophore signals were enhanced by immunostainings with respective antibodies. Magnifications of selected areas (*boxes*) are shown as insets. Scale bars in overviews: 10 µm; in magnifications: 2 µm. *C*, Manders’ colocalization coefficients of SorLA CT-GFP and SorLA Internal-mVenus with tdTomato-PICK1 (*top*) or tdTomato-GGA2 (*bottom*). Values represent mean ± SEM. Statistical differences were validated through Mann–Whittney U tests. Manders’ coefficient of SorLA CT-GFP/tdTomato-PICK1 (n = 7) is significantly lower than SorLA Internal-mVenus/tdTomato-PICK1 (n = 7; *p* = 0.0041). Manders’ coefficient of tdTomato-PICK1/SorLA CT-GFP is significantly lower than tdTomato-PICK1/SorLA Internal-mVenus (*p* = 0.0152). Manders’ coefficient of SorLA CT-GFP/tdTomato-GGA2 (n = 8) is significantly lower than SorLA Internal-mVenus/tdTomato-GGA2 (n = 7; *p* = 0.0059). n represents the number of cells analyzed from at least 2 wells. ∗, *p* ≤ 0.05; ∗∗, *p* ≤ 0.01.

We aimed at tagging each member of the Vps10p-D receptor family with fluorophores to investigate their dynamic localization by live imaging. We tagged SorLA at its C terminus with GFP (SorLA CT-GFP) and assessed colocalization with the interacting cytosolic adaptors PICK1 or GGA2 tagged with tdTomato. We observed low colocalization with PICK1 and limited colocalization with GGA2 (Fig. S1*A*). We reasoned that a C-terminal tag would impede interaction and colocalization with certain adaptor proteins. Therefore, we excluded C-terminal positioning of fluorophore tags. Instead, we designed expression constructs encoding receptors tagged internally in the luminal/extracellular moieties. The luminal/extracellular segments of Sortilin, SorCS1, SorCS2, and SorCS3 and the Vps10p-D of SorLA have been previously expressed in eukaryotic cells, purified from cell culture media, and successfully used in functional ligand-binding experiments (33, 34, 35, 37, 43). This demonstrated that these segments are functional units. In addition, extracellular moieties of Sortilin, SorLA, and SorCS2 have been also expressed to resolve their structures (28, 29, 30). Considering these results, we predicted functional segments after which fluorophore tags could be inserted without altering receptor functionality. We designed respective full-length receptors with mVenus or tdTomato tag insertions between the luminal/extracellular moieties and the transmembrane domains of Sortilin, SorCS1b, SorCS1c-α, SorCS2, and SorCS3 (Fig. 1*A*). For SorLA, a tag between the transmembrane domain and the fibronectin type III repeats constantly revealed errors in protein folding predictions, and thus, tag insertions were positioned directly after the SorLA Vps10p-D (Fig. 1*A*).

We predicted correct folding of the designed proteins computationally using AlphaFold 2.0 (70). Figure 1*B* shows that transmembrane domains (indicated in *orange*) of Sortilin, SorCS1, SorCS2, and SorCS3 fold as full α-helices in wt proteins and after insertion of either mVenus or tdTomato. The TopPred tool also recognized these transmembrane domains, respectively (71, 72). The leucine-rich domains (indicated in *green*) of the SorCS subgroup preceding the respective tag show the same folding sequence of α-helices and β-sheets as in the wt proteins (Fig. 1*B*). In tagged SorLA, each fluorophore is followed by an YWTD β-propeller domain that folds identical to the domain in wt SorLA (indicated in *dark blue*). These predictions indicate that the structures of the domains next to the fluorophores in the internally tagged Vps10p-D receptors are unaltered by mVenus or tdTomato insertions at the selected positions as compared with the wt receptors.

### Expression of internally tagged Vps10p-D receptors

**Figure 2:**
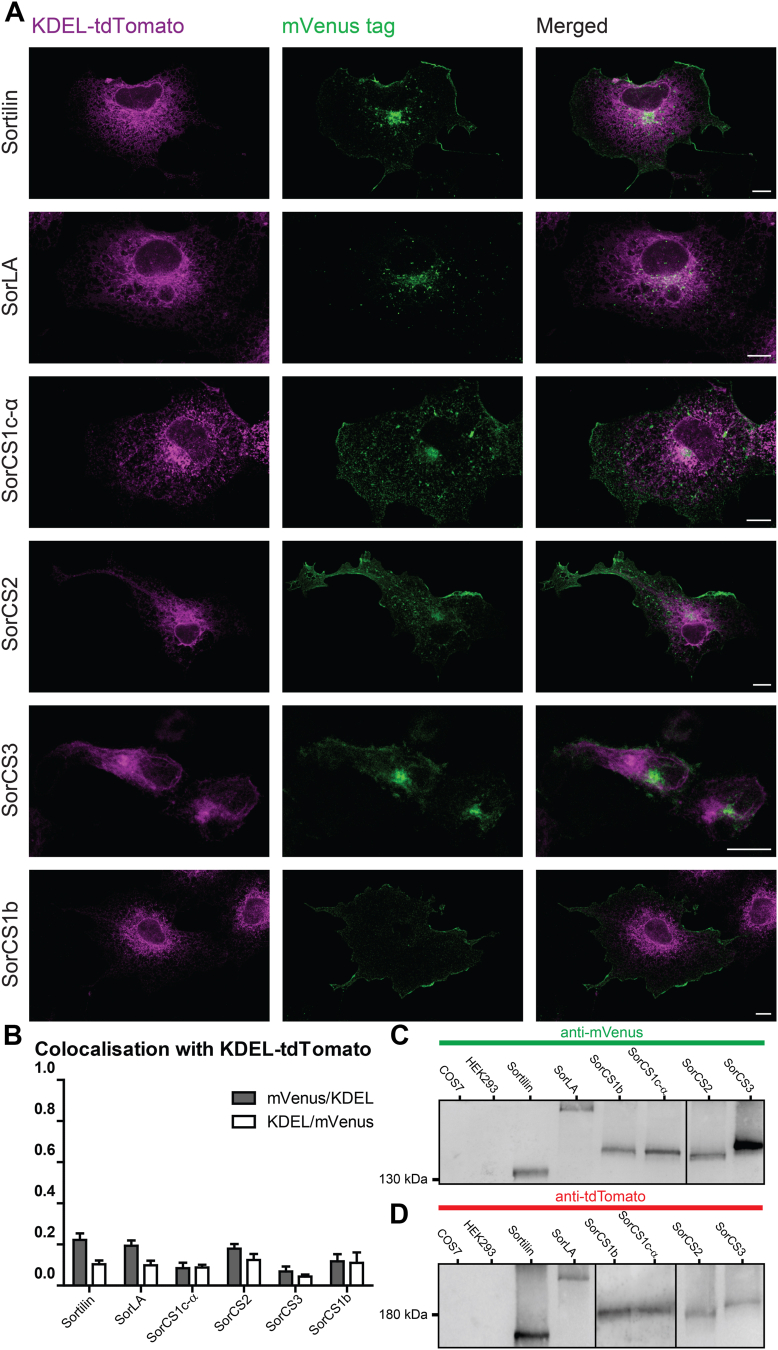
**Expression of the tagged Vps10p-D receptors.***A*, confocal images of cells transfected with the ER marker KDEL-tdTomato- (*magenta*) and mVenus-tagged Vps10p-D receptors (*green*). mVenus-tagged Sortilin, SorLA, SorCS1b, SorCS1c-α, and SorCS2 were expressed in COS7 cells, and mVenus-tagged SorCS3 was expressed in HEK293 cells. mVenus and tdTomato signals were enhanced by immunostainings with respective antibodies. Scale bars represent 10 μm. *B*, Manders’ coefficients of colocalization between KDEL-tdTomato and Sortilin-mVenus (n = 7), SorLA-mVenus (n = 8), SorCS1c-α-mVenus (n = 10), SorCS2-mVenus (n = 7), SorCS3-mVenus (n = 10), or SorCS1b-mVenus (n = 5). Values represent mean ± SEM. n represents the number of cells analyzed from at least two wells. *C* and *D*, full-length expression of fluorophore-tagged Vps10p-D receptors. Lysates of indicated nontransfected cells and COS7 cells transfected with mVenus- (*C*) or tdTomato- (*D*) tagged Sortilin, SorLA, SorCS1b, SorCS1c-α, and SorCS2 and HEK293 cells transfected with mVenus- (*C*) or tdTomato- (*D*) tagged SorCS3 were analyzed by immunoblotting with respective antibodies for (*C*) mVenus or (*D*) tdTomato. Data are representative of three independent experiments. ER, endoplasmic reticulum; HEK293, human embryonic kidney 293 cell line; Vps10p-D, Vps10p-domain.

**Fig S2:**
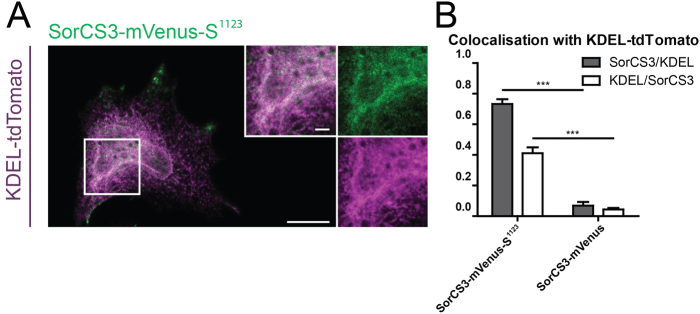
**Misplaced fluorophore tag impedes ER exit of SorCS3.** *A*, colocalization analysis of SorCS3 with an internal mVenus tag N-terminal of S^1123^ (SorCS3-mVenus-S^1123^) with KDEL-tdTomato. Confocal images of cells transfected with SorCS3-mVenus-S^1123^ (*green*) and KDEL-tdTomato (*red*). Fluorophore signals were enhanced by immunostainings with respective antibodies. Magnification of a selected area (*box*) are shown as insets. Scale bars in overviews: 10 µm; in magnifications: 2 µm. *B*, colocalization analysis of SorCS3-mVenus-S^1123^ and KDEL-tdTomato (confocal image shown in *A*) in comparison to SorCS3 with an internal mVenus tag N-terminal of P^1114^ (SorCS3-mVenus) and KDEL-tdTomato (confocal image shown in Fig. 2*A*). Manders’ coefficient of SorCS3-mVenus-S^1123^/KDEL-tdTomato (n = 7) is significantly higher than that of SorCS3-mVenus/KDEL-tdTomato (n = 10; *p* = 0.0008). Values represent mean ± s.e.m. Statistical differences were validated through Mann–Whittney U tests. n represents the number of cells analyzed from at least 2 wells. ∗∗∗, *p* ≤ 0.001.

Correctly folded proteins are exported from the endoplasmic reticulum (ER), whereas misfolded proteins are retained and eventually degraded. We cloned and expressed the designed Vps10p-D receptors tagged with mVenus or tdTomato in COS7 cells. Cotransfection of the mVenus constructs with an ER marker construct (KDEL-tdTomato) demonstrates low localization of the mVenus-tagged Vps10p-D receptors at the ER (Fig. 2, *A* and *B*). However, fluorophore insertion at some sites in SorCS3 resulted in constructs trapped in the ER (Fig. S2). Moreover, the final fluorophore-tagged SorCS3 construct was expressed in human embryonic kidney 293 (HEK293) cells because in COS7 cells, varying amounts of the construct were constantly trapped in the ER. For mVenus-tagged Sortilin, SorCS1c-α, SorCS2, and SorCS3, vesicular and surface expression was observed (Fig. 2*A*). SorLA-mVenus predominated in vesicular structures, and SorCS1b-mVenus mainly localized at the cell surface. Together, these findings indicate the successful exit of the tagged Vps10p-D receptors from the ER. Notably, we observed colocalization of internally mVenus-tagged SorLA with tdTomato-PICK1 and increased colocalization with tdTomato-GGA2 as compared with SorLA tagged at its C terminus (Fig. S1, *B* and *C*).

Immunoblotting of cell lysates with tag-specific antibodies revealed full-length expression of the mVenus- or the tdTomato-tagged receptors, as observed estimated molecular weights resemble the expected calculated molecular weights of the respective proteins (Fig. 2, *C* and *D*).

### Subcellular localization of internally tagged Vps10p-D receptors

**Figure 3:**
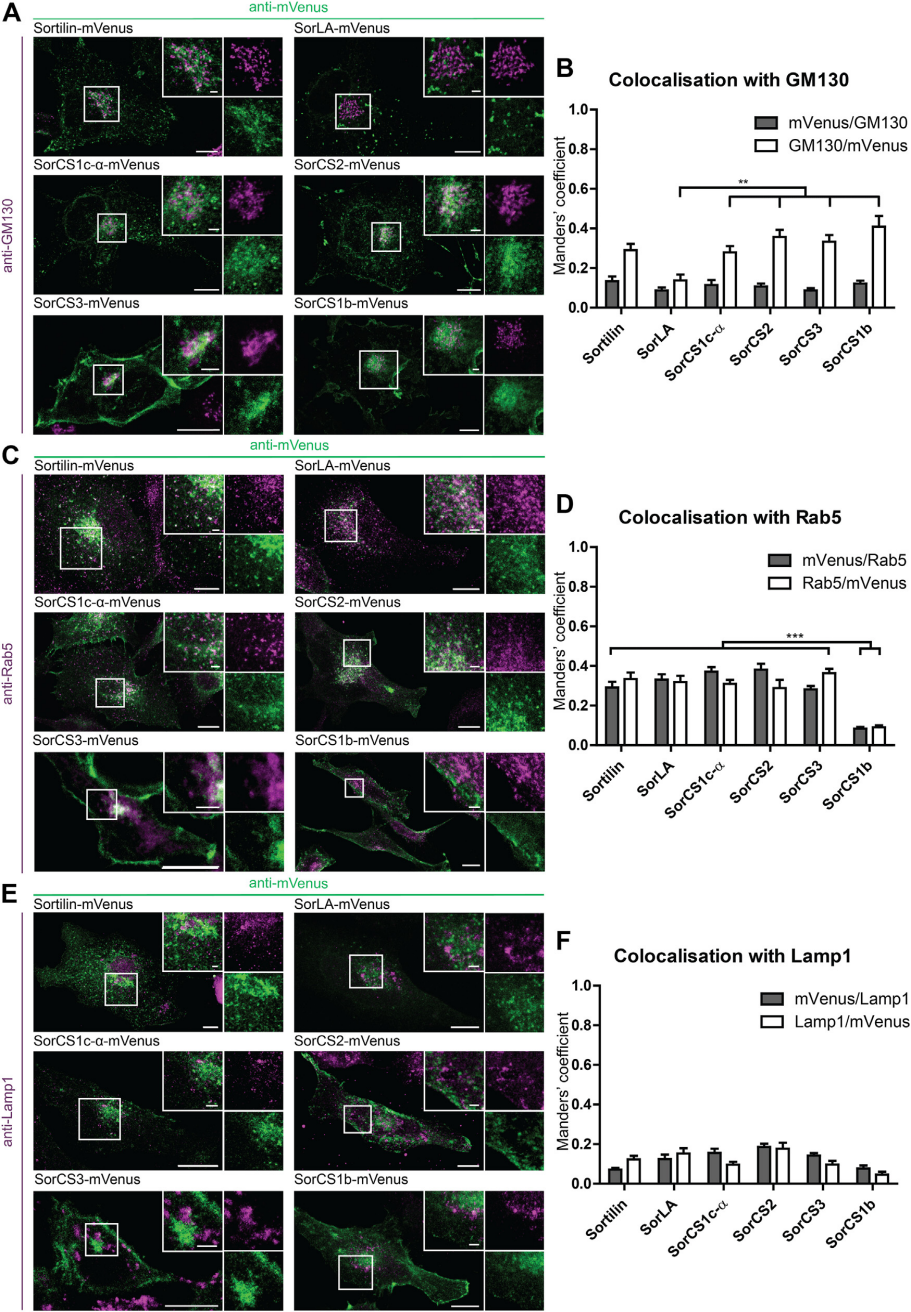
**Colocalization analysis of mVenus-tagged Vps10p-D receptors with endogenous GM130, Rab5, or LAMP1.***A*, *C*, and *E*, mVenus-tagged Sortilin, SorLA, SorCS1b, SorCS1c-α, and SorCS2 were expressed in COS7 cells, and mVenus-tagged SorCS3 was expressed in HEK293 cells, immunostained for endogenous (*A*) GM130, (*C*) Rab5, or (*E*) LAMP1 (*magenta*), mVenus signals (*green*) were enhanced by immunostaining, and cells were analyzed by confocal microscopy. Magnifications of selected areas (*boxes*) are shown as *insets*. Colocalization appears *white*. *B*, *D*, and *F*, Manders’ colocalization coefficients of the Vps10p-D receptors tagged with mVenus and (*B*) GM130, (*D*) Rab5, or (*F*) LAMP1. Values represent mean ± SEM. Statistical differences were validated through Kruskal–Wallis and post hoc Mann–Whitney *U* tests. *B*, Manders’ coefficient of GM130/SorLA-mVenus (n = 9) is significantly lower than GM130/Sortilin-mVenus (n = 14; *p* = 0.003), GM130/SorC1c-α-mVenus (n = 10; *p* = 0.008), GM130/SorCS2-mVenus (n = 10; *p* = 0.001), GM130/SorCS3-mVenus (n = 16; *p* ≤ 0.001), and GM130/SorC1b-mVenus (n = 11; *p* = 0.001). *D*, Manders’ coefficient of SorCS1b-mVenus/Rab5 (n = 19) is significantly lower than Sortilin-mVenus/Rab5 (n = 13; *p* ≤ 0.001), SorLA-mVenus/Rab5 (n = 10; *p* ≤ 0.001), SorCS1c-α-mVenus/Rab5 (n = 14; *p* ≤ 0.001), SorCS2-mVenus/Rab5 (n = 7; *p* ≤ 0.001), and SorCS3-mVenus/Rab5 (n = 24; *p* ≤ 0.001). Manders’ coefficient of Rab5/SorCS1b-mVenus (*p* ≤ 0.001) is significantly lower than Rab5/Sortilin-mVenus (*p* ≤ 0.001), Rab5/SorLA-mVenus (*p* ≤ 0.001), Rab5/SorCS1b-mVenus (*p* ≤ 0.001), Rab5/SorCS1c-α-mVenus (*p* ≤ 0.001), Rab5/SorCS2-mVenus (*p* ≤ 0.001), and Rab5/SorCS3-mVenus (*p* ≤ 0.001). *F*, Manders’ coefficients that form colocalization between LAMP1 and Sortilin-mVenus (n = 13), SorLA-mVenus (n = 12), SorCS1b-mVenus (n = 9), SorCS1c-α-mVenus (n = 12), SorCS2-mVenus (n = 12), or SorCS3-mVenus (n = 14) were all similarly low. n represents the number of cells analyzed from at least two wells. ∗∗*p* ≤ 0.01; ∗∗∗*p* ≤ 0.001. Scale bars in overviews represent 10 μm; in magnifications represent 2 μm. HEK293, human embryonic kidney 293 cell line; LAMP1, lysosomal-associated membrane protein 1; Vps10p-D, Vps10p-domain.

**Figure 4:**
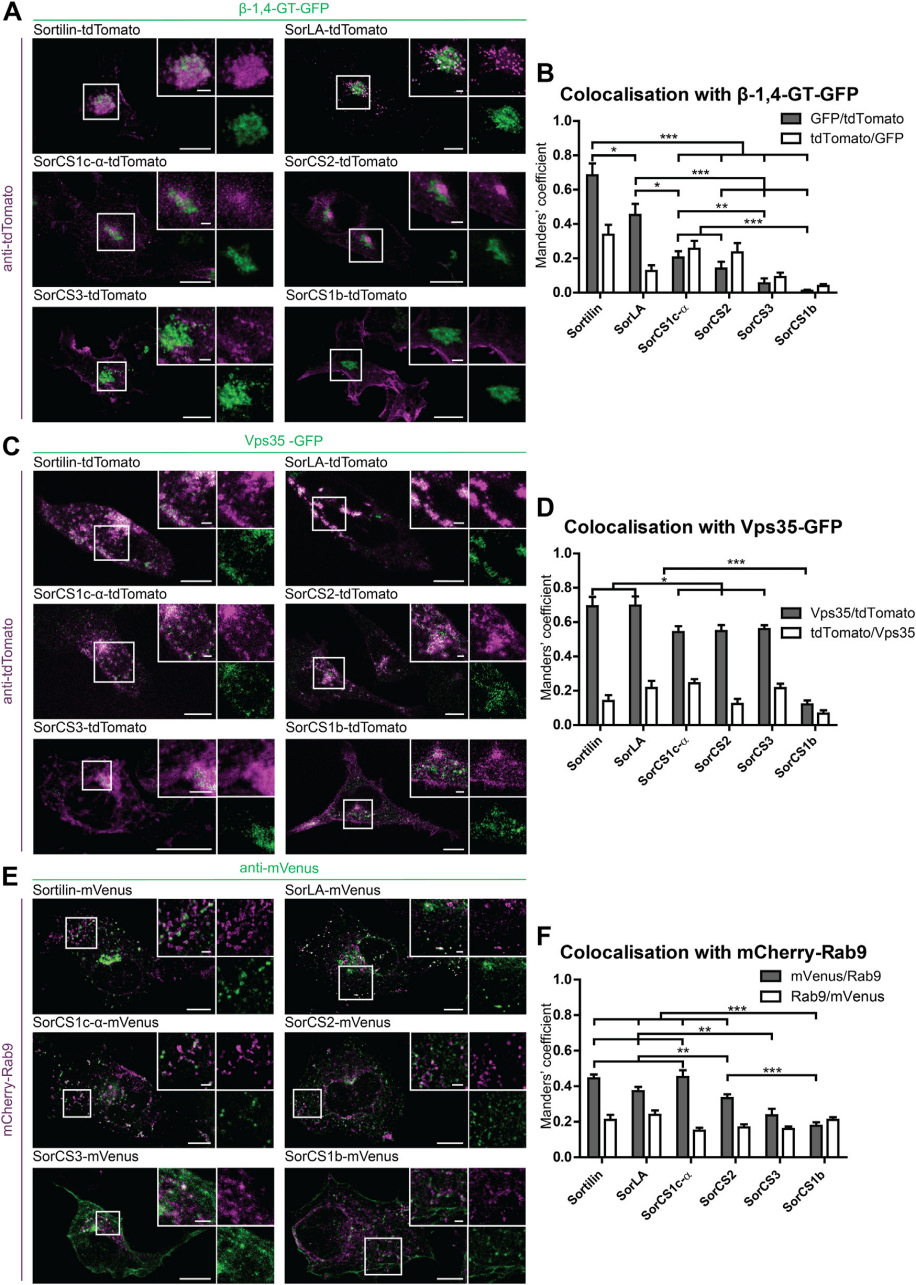
**Colocalization analysis of fluorophore-tagged Vps10p-D receptors with β-1,4-GT-GFP, Vps35-GFP, and mCherry-Rab9.***A*, *C*, and *E*, confocal images of cells transfected with Vps10p-D receptors tagged with (*A* and *C*) tdTomato (*magenta*) or (*E*) mVenus (*green*) and (*A*) β-1,4-GT-GFP, (*C*) Vps35-GFP, or (*E*) mCherry-Rab9. Tagged Sortilin, SorLA, SorCS1b, SorCS1c-α, and SorCS2 were expressed in COS7 cells and tagged SorCS3 was expressed in HEK293 cells. Fluorophore signals were enhanced by immunostainings with respective antibodies. Magnifications of selected areas (*boxes*) are shown as *insets*. Colocalization appears *white*. *B*, *D*, and *F*, Manders’ colocalization coefficients for the fluorophore-tagged Vps10p-D receptors with (*B*) β-1,4-GT-GFP, (*D*) Vps35-GFP, or (*F*) Rab9-mCherry. Values represent mean ± SEM. Statistical differences were validated through Kruskal–Wallis and post hoc Mann–Whitney *U* tests. *B*, Manders’ coefficient of β-1,4-GT-GFP/Sortilin-tdTomato (n = 11) is significantly higher than β-1,4-GT-GFP/SorLA-tdTomato (n = 11; *p* = 0.028), β-1,4-GT-GFP/SorCS1c-α-tdTomato (n = 10; *p* ≤ 0.001), β-1,4-GT-GFP/SorCS2-tdTomato (n = 9; *p* ≤ 0.001), β-1,4-GT-GFP/SorCS3-tdTomato (n = 11; *p* ≤ 0.001), and β-1,4-GT-GFP/SorCS1b-tdTomato (n = 22; *p* ≤ 0.001). Manders’ coefficient of β-1,4-GT-GFP/SorLA-tdTomato is significantly higher than β-1,4-GT-GFP/SorCS1c-α-tdTomato (*p* = 0.013), β-1,4-GT-GFP/SorCS2-tdTomato (*p* = 0.001), β-1,4-GT-GFP/SorCS3-tdTomato (*p* ≤ 0.001), and β-1,4-GT-GFP/SorCS1b-tdTomato (*p* ≤ 0.001). Manders’ coefficient of β-1,4-GT-GFP/SorCS1c-α-tdTomato is significantly higher than β-1,4-GT-GFP/SorCS3-tdTomato (*p* = 0.002) and β-1,4-GT-GFP/SorCS1b-tdTomato (*p* ≤ 0.001). Manders’ coefficient of β-1,4-GT-GFP/SorCS2-tdTomato is significantly higher than β-1,4-GT-GFP/SorCS1b-tdTomato (*p* ≤ 0.001). *D*, Manders’ coefficient of Vps35-GFP/SorCS1b-tdTomato (n = 10) is significantly lower than Vps35-GFP/Sortilin-tdTomato (n = 14; *p* ≤ 0.001), Vps35-GFP/SorLA-tdTomato (n = 10; *p* ≤ 0.001), Vps35-GFP/SorCS1c-α-tdTomato (n = 15; *p* ≤ 0.001), Vps35-GFP/SorCS2-tdTomato (n = 12; *p* ≤ 0.001), and Vps35-GFP/SorCS3-tdTomato (n = 13; *p* ≤ 0.001). Manders’ coefficients of Vps35-GFP/Sortilin-tdTomato (n = 14) and Vps35-GFP/SorLA-tdTomato (n = 10) significantly higher than Vps35-GFP/SorCS1c-α-tdTomato (n = 15; p_Sortilin =_ 0.029; p_SorLA_ = 0.031), Vps35-GFP/SorCS2-tdTomato (n = 12; p_Sortilin_ = 0.041; p_SorLA_ = 0.043), and Vps35-GFP/SorCS3-tdTomato (n = 13; p_Sortilin_ = 0.048; p_SorLA_ = 0.042). *F*, Manders’ coefficient of SorCS1b-mVenus/Rab9-mCherry (n = 11) is significantly lower than Sortilin-mVenus/mCherry-Rab9 (n = 18; *p* ≤ 0.001), SorLA-mVenus/mCherry-Rab9 (n = 16; *p* ≤ 0.001), SorCS1c-α-mVenus/mCherry-Rab9 (n = 10; *p* ≤ 0.001), and SorCS2-mVenus/mCherry-Rab9 (n = 12; *p* ≤ 0.001). Manders’ coefficient of SorCS2-mVenus/mCherry-Rab9 is significantly lower than Sortilin-mVenus/mCherry-Rab9 (*p* ≤ 0.001) and SorCS1c-α-mVenus/mCherry-Rab9 (*p* = 0.004). Manders’ coefficient of SorCS3-mVenus/mCherry-Rab9 (n = 12) is significantly lower than Sortilin-mVenus/mCherry-Rab9 (*p* ≤ 0.001), SorLA-mVenus/mCherry-Rab9 (*p* = 0.006), and SorCS1c-α-mVenus/mCherry-RAB9 (*p* ≤ 0.001). n represents the number of cells analyzed from at least two wells. ∗*p* ≤ 0.05; ∗∗*p* ≤ 0.01; ∗∗∗*p* ≤ 0.001. Scale bars in overviews represent 10 μm; in magnifications represent 2 μm. HEK293, human embryonic kidney 293 cell line; Vps10p-D, Vps10p-domain.

Next, we asked to which specific subcellular compartments the tagged receptors localize and if these localizations are in accordance with the previously reported localization of the Vps10p-D receptors. Therefore, the subcellular localizations of the mVenus- or tdTomato-tagged Vps10p-D receptors were specified by their expression and costaining of transfected cells with antibodies against subcellular marker proteins for the cis-Golgi network (CGN), early endosomes, or lysosomes (Fig. 3). In addition, we coexpressed fluorophore-tagged marker proteins for the TGN, the TGN–endosome transition, or endosome–lysosome transition (Fig. 4). Subsequently, Mander's colocalization coefficients were calculated from confocal images of single cells and compared between the sorting receptors. mVenus-tagged Sortilin, SorCS1c-α, SorCS2, SorCS3, and SorCS1b showed colocalization with the CGN marker protein GM130 (Fig. 3, *A* and *B*). For SorLA-mVenus, however, we observed significantly lower colocalization with GM130. Antibody staining against the early endosomal protein Rab5 showed significantly higher colocalization with mVenus-tagged Sortilin, SorLA, SorCS1c-α, SorCS2, and SorCS3 than with SorCS1b-mVenus (Fig. 3, *C* and *D*). The mVenus-tagged Vps10p-D receptors showed relatively low colocalization with the lysosomal protein LAMP1 (lysosomal-associated membrane protein 1) without significant differences between the receptors (Fig. 3, *E* and *F*). Coexpression of the GFP-tagged C-terminal fragment of the TGN-localized β-1,4-galactosyltransferase with tdTomato-tagged Vps10p-D receptors demonstrated high colocalization with Sortilin-tdTomato and SorLA-tdTomato (Fig. 4, *A* and *B*). Whereas SorCS1c-α-, SorCS2-, and SorCS3-tdTomato showed significantly lower colocalization with the TGN marker, and almost no colocalization was observed with SorCS1b-tdTomato. Vps35 is part of the retromer complex, which conveys endosome-to-TGN retrieval of different cargo proteins (73) and, hence, localizes to endosomes and the TGN. Vps35-GFP colocalized to a minor degree with SorCS1b-tdTomato (Fig. 4, *C* and *D*) but revealed significant colocalization with tdTomato-tagged SorCS1c-α, SorCS2, and SorCS3 and even higher colocalization with Sortilin- and SorLA-tdTomato (Fig. 4, *C* and *D*). Rab9 mediates transport from late endosomes to the TGN and toward endolysosomal compartments (74). mCherry-Rab9 colocalized with mVenus-tagged Sortilin, SorLA, SorCS1c-α, SorCS2, and only to a limited degree with SorCS3- and SorCS1b-mVenus (Fig. 4, *E* and *F*).

**Figure 5:**
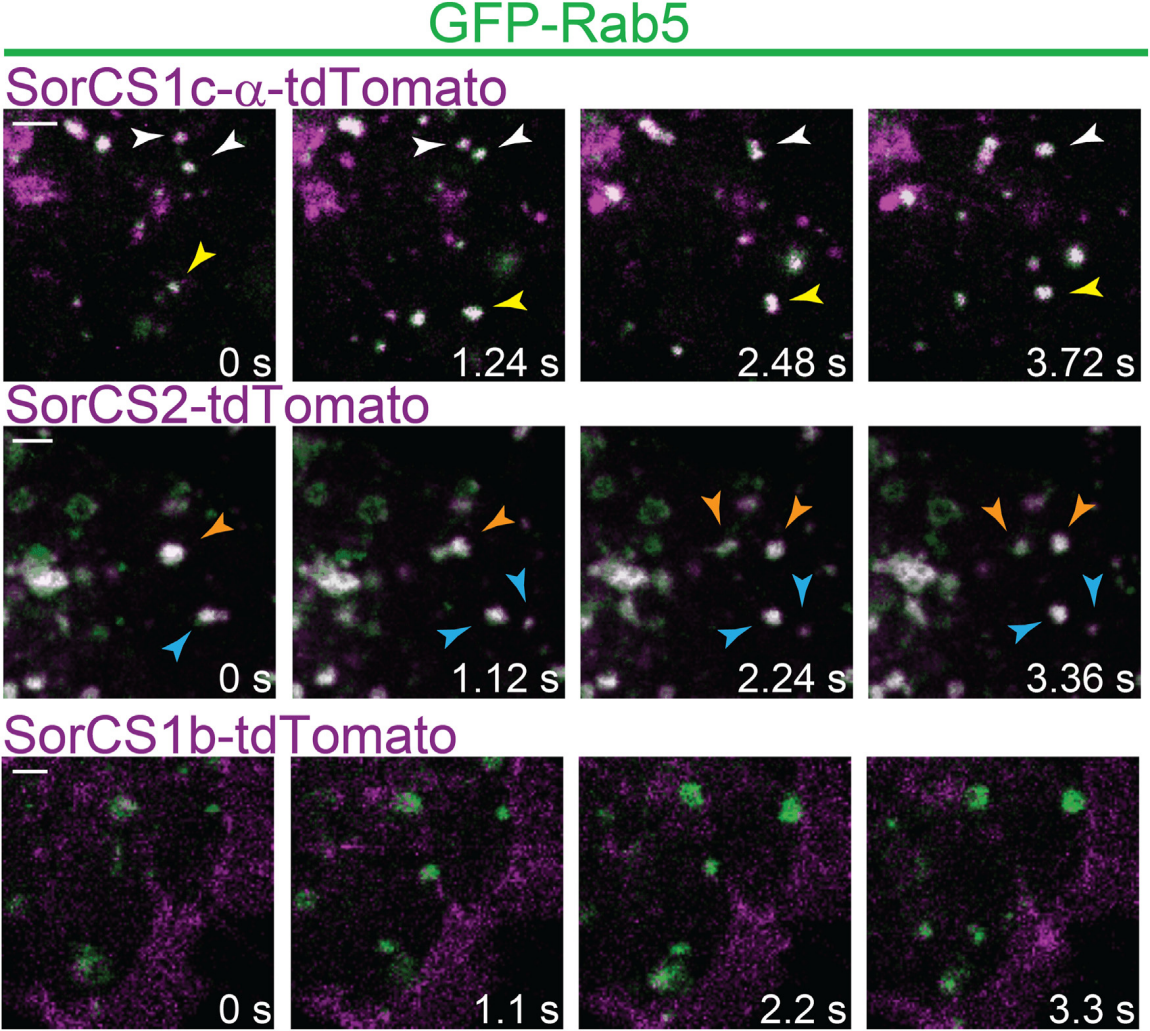
**Cotransport of tdTomato-tagged Vps10p-D receptors with GFP-Rab5**. Confocal live image analysis of COS7 cells transfected with SorCS1c-α-tdTomato, SorCS2-tdTomato, or SorCS1b-tdTomato (*magenta*) and GFP-Rab5 (*green*). Colocalization appears *white*. Single images show initial position (0 s) and after indicated time points. Different *arrows* indicate different mobile endosome fusion and fission events. The images correspond to Movies S3, S4 and S6. Scale bars represent 2 μm. Vps10p-D, Vps10p-domain.

**Figure 6:**
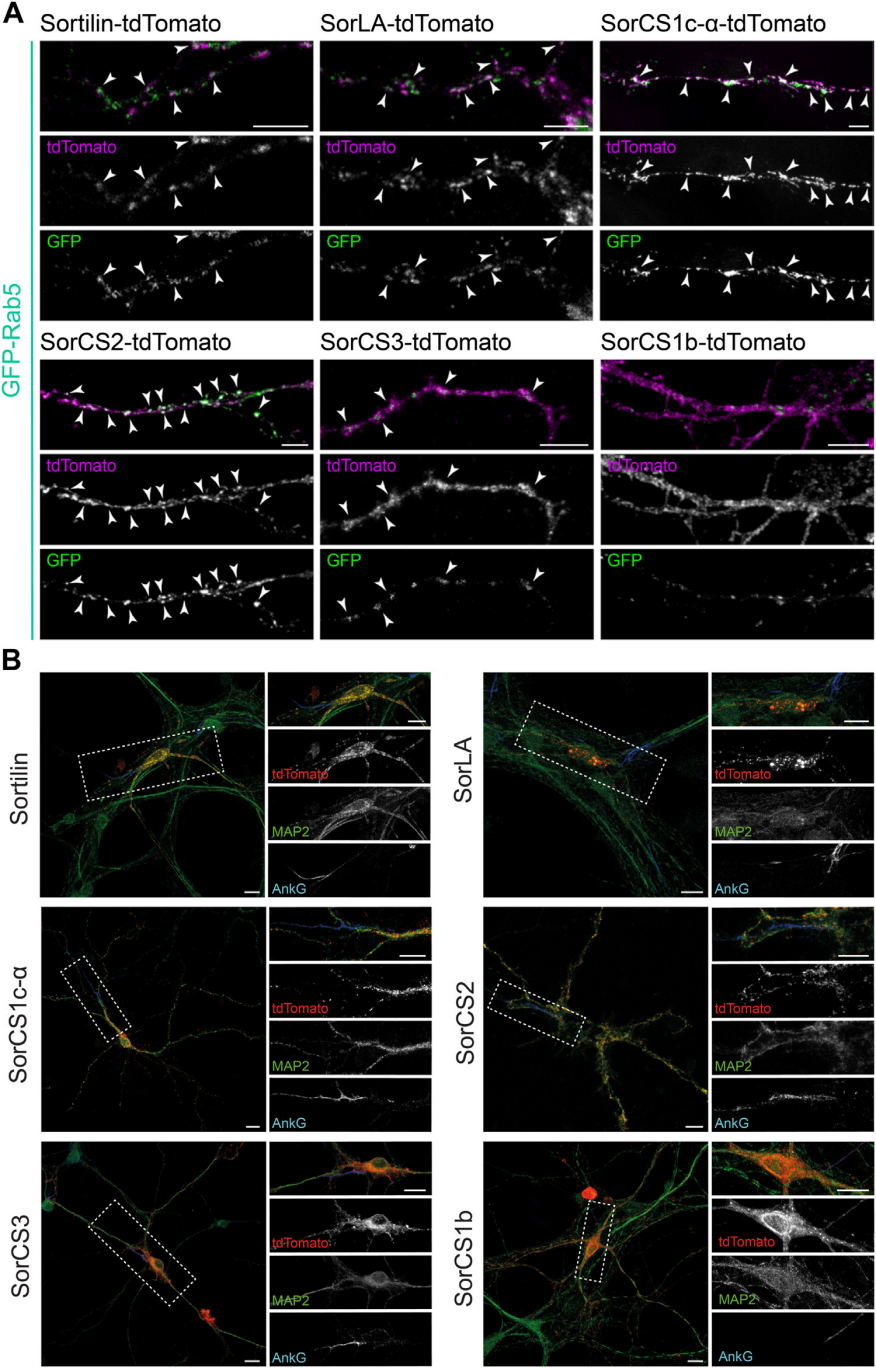
**Endosomal and somatodendritic localization of fluorophore-tagged Vps10p-D receptors in neurons.***A*, dissociated hippocampal neurons were transfected at 3 days *in vitro* (DIV 3) with the indicated tdTomato-tagged Vps10p-D receptors and GFP-Rab5 and immunostained at 7 to 14 DIV and analyzed by confocal microscopy. GFP (*green*) and tdTomato (*magenta*) signals were enhanced by immunostainings with respective antibodies. Colocalization appears *white*. Scale bars represent 5 μm. *B*, dissociated hippocampal neurons were transfected at DIV 3 with the indicated tdTomato-tagged Vps10p-D receptors (*red*) and immunostained for MAP2 (*green*), as a marker for dendrites, and for ankyrin G (AnkG, *blue*), as a marker for the axonal initial segment at DIV 7 to 14. Magnifications of selected areas (*boxed areas*) are shown on the *right*. Scale bars represent 10 μm. MAP2, microtubule-associated protein 2; Vps10p-D, Vps10p-domain.

In addition, COS7 or HEK293 cells and primary hippocampal neurons were transfected with GFP-Rab5 and the tdTomato-tagged Vps10p-D receptors. Expression in COS7 and HEK293 cells was monitored *in vivo* by confocal live-cell imaging. Time-lapse recordings demonstrate frequent endosomal cotransport of tdTomato-tagged Sortilin, SorLA, SorCS1c-α, SorCS2, and SorCS3 with GFP-Rab5 (Movies S1–S5 and Fig. 5). SorCS1b-tdTomato-positive vesicular structures were only rarely positive for GFP-Rab5 (Movie S6 and Fig. 5). Transfected primary hippocampal neurons were stained for GFP and tdTomato. A large number of vesicles positive for tdTomato-tagged Sortilin, SorLA, SorCS1c-α, SorCS2, and SorCS3 were also positive for GFP-Rab5 (Fig. 6*A*). In accordance with the previous experiments, we observed almost no colocalization of the surface-localized SorCS1b-tdTomato with GFP-Rab5. Costaining of primary cultured neurons expressing td-Tomato-tagged Sortilin, SorLA, SorCS1b, SorCS1c-α, SorCS2, or SorCS3 with the somatodendritic marker microtubule-associated protein 2 (MAP2) and the axonal initial segment marker ankyrin G demonstrated a predominant somatodendritic localization of all tagged Vps10p-D receptors (Fig. 6*B*). However, tagged SorCS3 and SorCS1b also showed some expression in the axonal initial segment.

Taken together, we observed somatodendritic and endosomal expression of all receptors except for SorCS1b, which presented a predominant cell surface localization, in neurons. All tagged Vps10p-D receptors, but not SorCS1b, showed an endosomal localization also in nonpolarized cells. Tagged Sortilin, SorLA, SorCS1c-α, SorCS2, and SorCS3 were all expressed in early to late endosomes, including Rab5-, Vps35-, and Rab9-positive endosomes. SorCS3, however, was found only to a minor extent in Rab9-positive endosomes. The subcellular localization has not been completely described for all Vps10p-D receptors, but our findings are to a large extent in accordance with so far reported localizations.

### Dimerization of SorCS2

**Figure 7:**
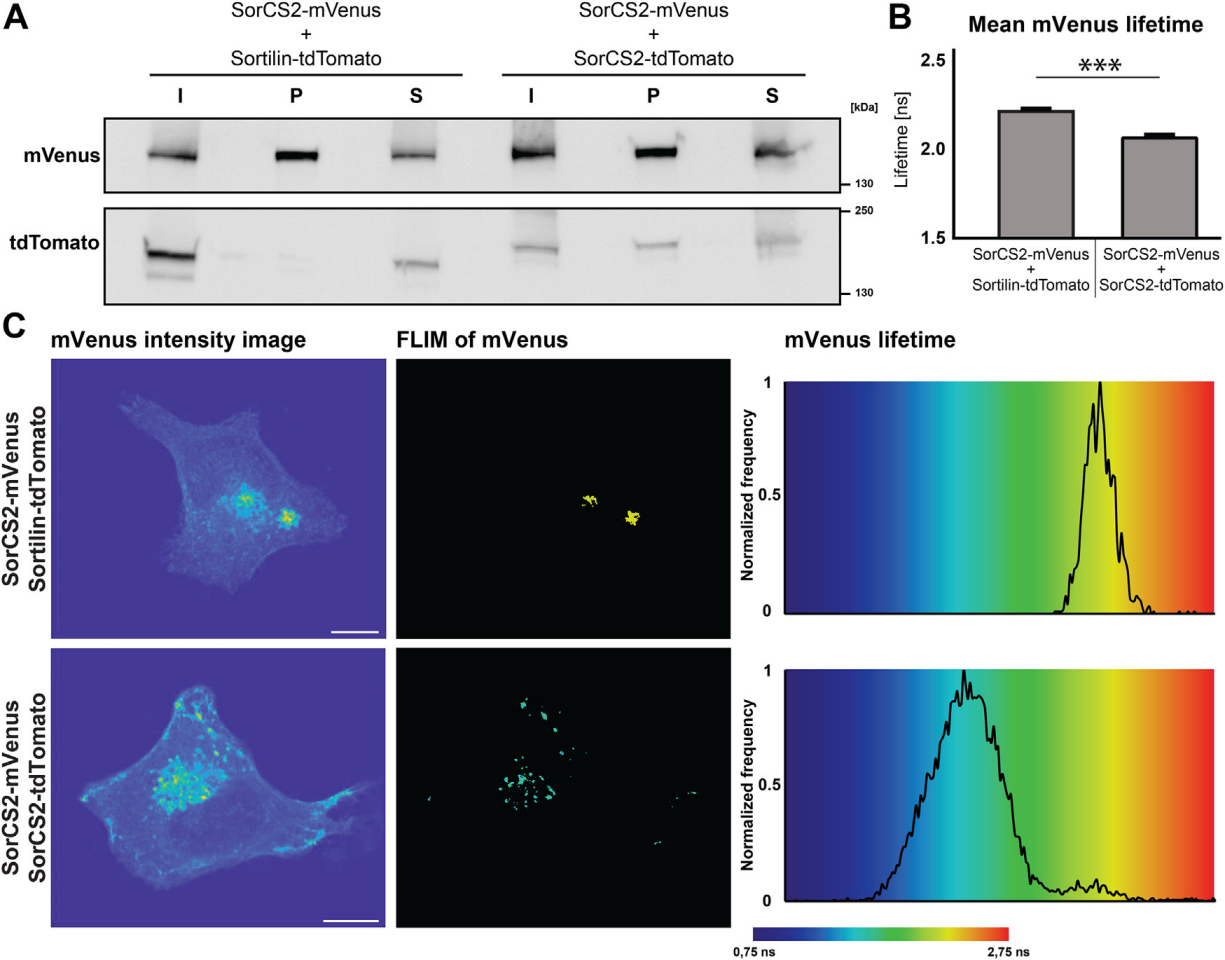
**Dimerization of SorCS2-mVenus with SorCS2-tdTomato but not with Sortilin-tdTomato.***A*, HeLa cells were transfected with SorCS2-mVenus and Sortilin-tdTomato or SorCS2-tdTomato as indicated. SorCS2-mVenus was precipitated from cell lysates with anti-mVenus beads, and fractions were analyzed by immunoblotting for the indicated fluorophore tags. I, input; P, precipitate; and S, supernatant. The depicted immunoblot is representative out of three independent experiments. *B* and *C*, FLIM analysis of HeLa cells transfected with SorCS2-mVenus and Sortilin-tdTomato or SorCS2-tdTomato. *B*, mean ± SEM. Lifetimes of excited mVenus in pixels above an equal intensity threshold. mVenus lifetime is significantly lower in cells coexpressing SorCS2-tdTomato (n_cells =_ 25; n_pixels =_ 79,984) than in cells coexpressing Sortilin-tdTomato (n_cells_ = 25; n_pixels_ = 82,816; *p* ≤ 0.001). *C*, mVenus intensity image, intensity images of SorCS2-mVenus in cells coexpressing Sortilin-tdTomato or SorCS2-tdTomato (*left*), FLIM of mVenus, FLIM representation of excited mVenus in pixels above an equal intensity threshold (*middle*). Normalized frequency, lifetime distribution of pixels with acceptor excitation (*right*). mVenus lifetime is represented as indicated in the color bar. *Black pixels* represent pixels below the intensity threshold. Scale bars represent 10 μm. FLIM, fluorescence lifetime imaging microscopy.

The correct subcellular localization of the fluorophore-tagged Vps10p-D receptors and the therefore assumed functionality of their cytoplasmic tails interacting with different cytosolic adaptor proteins raised the intriguing question whether their extracellular domains are also functional. The leucine-rich domain of the SorCS subgroup interacts with other leucine-rich domains of the SorCS subgroup, which consequently form homodimers and heterodimers. As this domain is adjacent to the tag insert area in the SorCS subgroup, we regarded dimerization, which likely underlies functionality, as crucial. As a proof, we investigated the ability of tagged SorCS2 to form homodimers. To this end, we expressed mVenus- and tdTomato-tagged SorCS2 and performed co-IP experiments. As a control, these experiments were also performed with cells transfected with SorCS2-mVenus and Sortilin-tdTomato. Sortilin, which lacks a leucine-rich domain, has not been shown to dimerize with any member of the SorCS subgroup. We observed co-IP of SorCS2-tdTomato with SorCS2-mVenus but not of Sortilin-tdTomato with SorCS2-mVenus from the respective cell lysates (Fig. 7*A*).

Next, we assessed homodimerization of fluorophore-tagged SorCS2 by employing FLIM. Again, we coexpressed SorCS2-mVenus with either SorCS2-tdTomato or Sortilin-tdTomato. Cells were fixed, and the fluorescence lifetime of the mVenus fluorophores was measured using simulated emission depletion (STED) microscopy. Here, mVenus emission acts as a donor for the excitation of tdTomato. If both fluorophores are in close proximity, the fluorescence lifetime of mVenus decreases because of photon energy transfer to tdTomato. When coexpressing SorCS2-mVenus with SorCS2-tdTomato, the fluorescence lifetime of the mVenus fluorophore was significantly lower as compared with SorCS2-mVenus coexpressed with Sortilin-tdTomato (Fig. 7, *B* and *C*). This proves close proximity between the tagged SorCS2 proteins and strongly suggests their direct interaction and homodimerization.

### Vps10p-D receptors mediate BDNF uptake

**Figure 8:**
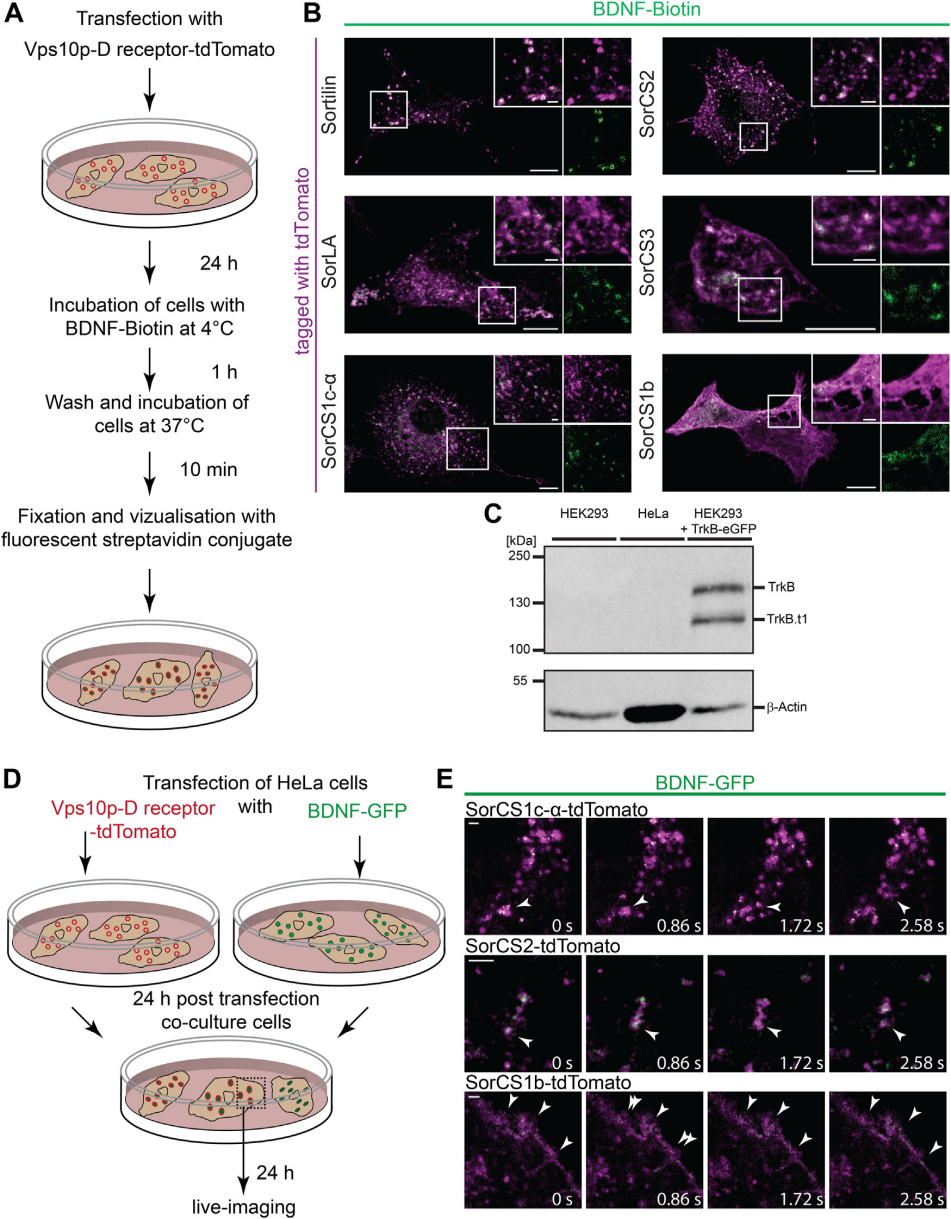
**Fluorophore-tagged Vps10p-D receptors mediate BDNF uptake.***A*, design of the BDNF–biotin uptake experiment shown in (*B*). *B*, tdTomato-tagged Sortilin, SorLA, SorCS1b, SorCS1c-α, and SorCS2 were expressed in HeLa cells, and tdTomato-tagged SorCS3 was expressed in HEK293 cells. Cells were incubated with BDNF–biotin for 1 h at 4 °C, washed, incubated for 10 min at 37 °C, and fixed. BDNF–biotin visualized by a fluorescent streptavidin conjugate (*green*) and the tdTomato signal (*magenta*) was enhanced by immunostaining. Magnifications of selected areas (*boxes*) of confocal images are shown as *insets*. Colocalization appears *white*. Scale bars in overviews represent 10 μm; in magnifications represent 2 μm. *C*, lysates of nontransfected HEK293 and HeLa cells as well as HEK293 cells transfected with TrkB-GFP were analyzed by immunoblotting with antibodies against TrkB (*upper panel*) and β-actin (*lower panel*). The immunoblot shows a representative image out of three independent experiments. *D*, design of the BDNF-GFP uptake experiment shown in *E* and Movies S7–S11. *E*, HeLa cells were transfected with tdTomato-tagged Vps10p-D receptors (*magenta*) and in parallel another set of HeLa cells was transfected with BDNF-GFP (*green*). After 24 h, cells were cocultured and imaged after 24 h by live confocal microscopy. Single images show selected areas of cells of initial recordings (0 s) and after indicated time points. Colocalization appears *white*. *Arrows* point to exemplary colocalization. The images correspond to Movies S9–S11. Scale bars represent 2 μm. BDNF, brain-derived neurotrophic factor; HEK293, human embryonic kidney 293 cell line; TrkB, tropomyosin receptor kinase B.

To analyze functional ligand binding of the fluorophore-tagged Vps10p-D receptors, we assessed BDNF-binding abilities of the Vps10p domains. Cells were transfected with the tdTomato-tagged Vps10p-D receptors, incubated at 4 °C with mature BDNF–biotin, washed, incubated for 10 min at 37 °C to allow receptor internalization, and after fixation, BDNF–biotin was visualized by a fluorescent streptavidin conjugate (Fig. 8*A*). HeLa or HEK293 cells transfected with tdTomato-tagged Sortilin, SorLA, SorCS1c-α, SorCS2, and SorCS3 internalized BDNF–biotin (Fig. 8*B*). The BDNF signal was restricted to vesicular structures that were also positive for the respective tagged Vps10p-D receptors. HeLa cells transfected with tdTomato-tagged SorCS1b yielded BDNF staining only at the cellular surface, and no internalization of BDNF was observed (Fig. 8*B*). We performed immunoblotting for the BDNF receptor TrkB. We compared lysates of HEK293 cells transfected with TrkB-GFP and nontransfected HeLa and HEK293 cells (Fig. 8*C*). Using an antibody against TrkB, we could not detect TrkB in the employed HeLa and HEK293 cells but in transfected cells. We detected TrkB-GFP with the expected molecular weight of approximately 167 kDa, and its dominant-negative truncated version TrkB.t1-GFP, which originates from post-translational cleavage of TrkB-GFP within its cytoplasmic domain by calpain (75) with an expected molecular weight of approximately 117 kDa.

We assessed BDNF internalization also by live-cell imaging by coculturing cells expressing BDNF-GFP with cells expressing tdTomato-tagged Sortilin, SorLA, SorCS1b, SorCS1c-α, or SorCS2. In these experiments, one set of HeLa cells was transfected with BDNF-GFP (Fig. 8*D*). These cells secrete BDNF-GFP into the cell culture medium. Another set of HeLa cells was transfected with the tdTomato-tagged Vps10p-D receptors. About 24 h after transfection, the differently treated cells were cocultured and analyzed by confocal live microscopy. Time-lapse images of cells expressing either of the tdTomato-tagged Vps10p-D receptors also show vesicular colocalization and cotransport of BDNF-GFP with tdTomato-tagged Sortilin, SorLA, SorCS1c-α, or SorCS2 (Movies S7–S10 and Fig. 8*E*). As BDNF-GFP is expressed in independently transfected cells, tdTomato-tagged Vps10p-D receptor–expressing cells took up BDNF-GFP from the cell culture medium. In accordance with the previous experiments, BDNF-GFP was observed predominantly at the cellular surface in cells expressing SorCS1b-tdTomato (Movie S11 and Fig. 8*E*).

**Figure 9:**
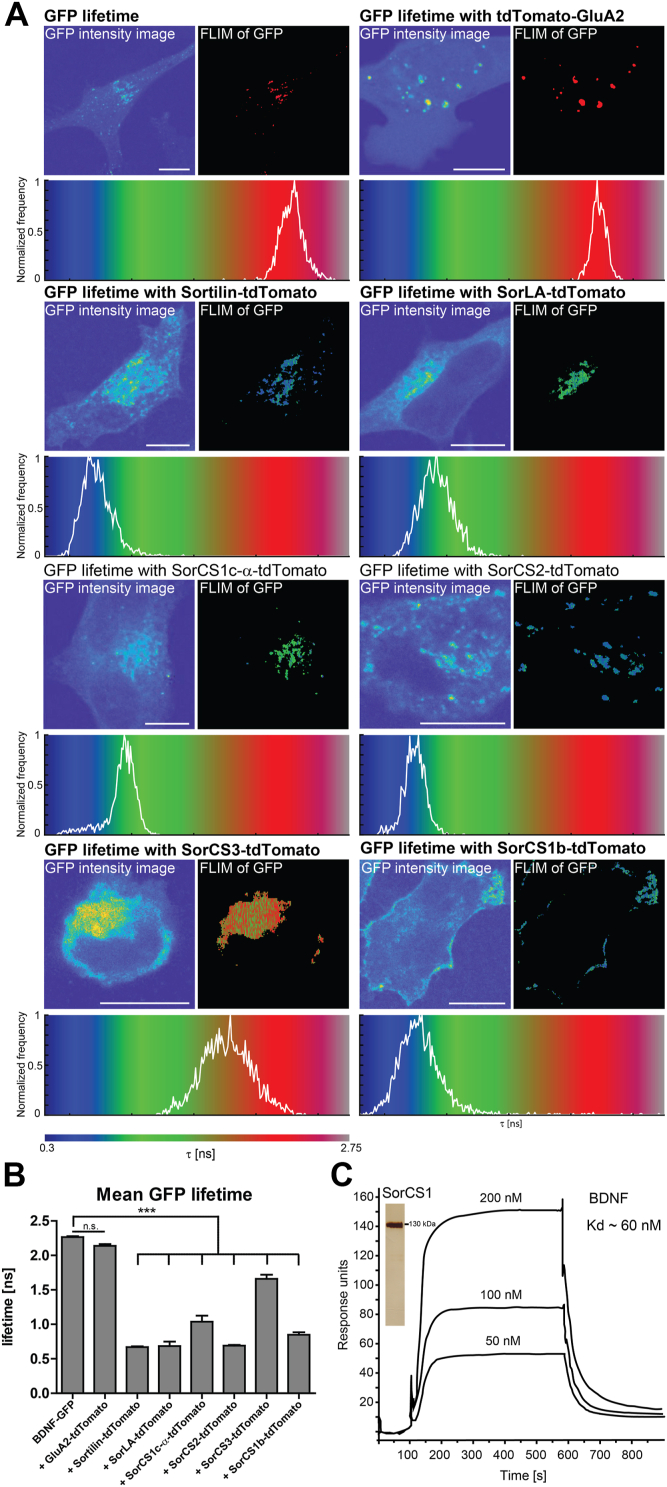
**Fluorophore-tagged Vps10p-D receptors interact with BDNF.***A* and *B*, FLIM analysis of HeLa cells expressing BDNF-GFP alone or cells transfected with tdTomato-tagged GluA2, Sortilin, SorLA, SorCS1c-α, SorCS2, SorCS3, or SorCS1b, which were cocultivated with HeLa cells expressing BDNF-GFP. tdTomato-tagged GluA2 was expressed in SH-SY5Y cells, tdTomato-tagged Sortilin, SorLA, SorCS1c-α, SorCS2, or SorCS1b in HeLa cells, and tdTomato-tagged SorCS3 in HEK293 cells. *A*, shown are for the respective cells as indicated: GFP intensity image, intensity images of BDNF-GFP (*left*), and FLIM of GFP, FLIM representation of excited GFP in pixels above an equal intensity threshold (*right*). GFP lifetime (l*ower panels*) corresponds to the color bar (*bottom*). *Black pixels* represent pixels below the intensity threshold. Normalized frequency, normalized lifetime distribution of pixels above an equal intensity threshold. Scale bars represent 10 μm. *B*, mean ± SEM. Lifetimes of excited GFP in pixels above an equal intensity threshold. GFP lifetime is significantly higher in cells expressing only BDNF-GFP (n_cells_ = 10; n_pixels =_ 19,740) than in cocultivated cells expressing tdTomato-tagged Sortilin (n_cells =_ 15; n_pixels =_ 47,964; *p* ≤ 0.0001), SorLA (n_cells =_ 15; n_pixels =_ 10,853; *p* ≤ 0.0001), SorCS1c-α (n_cells =_ 11; n_pixels =_ 11,277; *p* ≤ 0.0001), SorCS2 (n_cells =_ 15; n_pixels =_ 15,382; *p* ≤ 0.0001), SorCS3 (n_cells =_ 1 2; n_pixels =_ 18,082; *p* ≤ 0.0001), or SorCS1b (n_cells =_ 14; n_pixels =_ 27,801; *p* ≤ 0.0001) but not as in cells expressing tdTomato-tagged GluA2 (n_cells =_ 9; n_pixels =_ 25,204; *p* = 0.3638). ∗∗∗*p* ≤ 0.0001. *C*, binding of BDNF was measured by surface plasmon resonance analysis with 50 fmol/mm^2^ purified extracellular moiety of SorCS1 immobilized to a sensor chip. The *inset* shows purity of SorCS1 (silver-stained gel). The sensor chip was superfused with mature BDNF (50–200 nM) containing buffer at 100 s followed by buffer alone at 600 s. The calculated *K*_*d*_ value is indicated. BDNF, brain-derived neurotrophic factor; FLIM, fluorescence lifetime imaging microscopy; HEK293, human embryonic kidney 293 cell line; Vps10p-D, Vps10p-domain.

We monitored the interaction of the tdTomato-tagged Vps10p-D receptors and BDNF-GFP by FLIM. HeLa cells were transfected with BDNF-GFP and cultured alone or cocultured with cells transfected with the receptor constructs or a control (tdTomato-GluA2). The control construct, tdTomato-GluA2, encodes a signal peptide followed by tdTomato and the terminal transmembrane and cytosolic domains of GluA2, corresponding to a type I transmembrane protein with tdTomato as the extracellular moiety. We expressed this control construct in SH-SY5Y cells that, in contrast to HeLa or HEK293 cells, take up BDNF-GFP. Cells were fixed, and the fluorescence lifetime of GFP was measured using STED microscopy. Here, GFP emission acts as a donor for the excitation of tdTomato. If both fluorophores are in close proximity, the fluorescence lifetime of GFP decreases because of photon energy transfer to tdTomato. The fluorescence lifetime of GFP was almost identical in HeLa cells expressing BDNF-GFP cultured alone and in cocultured SH-SY5Y cells taken up BDNF-GFP and expressing tdTomato-GluA2 (Fig. 9, *A* and *B*). In contrast, the fluorescence lifetime of GFP in cocultured cells positive for BDNF-GFP and tdTomato-tagged Vps10p-D receptors was significantly lower than in HeLa cells expressing only BDNF-GFP- or tdTomato-GluA2-transfected cocultured SH-SY5Y cells (Fig. 9, *A* and *B*). To confirm direct interaction of BDNF and SorCS1, we purified the extracellular moiety of SorCS1 from stably transfected Chinese hamster ovary cells and analyzed BDNF binding by surface plasmon resonance analysis (Fig. 9*C*). BDNF binds to the purified ectodomain of SorCS1 with an estimated *K*_*d*_ of 60 nM.

## Discussion

Accurate sorting of proteins is one factor that underlies the compartmentalization of cells into distinct membrane-bound organelles and the asymmetric arrangement of polarized cells such as neurons. Alterations of these processes can underlie tremendous cytopathological defects. Sorting receptors of the Vps10p-D family play a role in endosomal targeting, secretion, and internalization of soluble ligands. In addition, they govern also the subcellular localization of other proteins, but the underlying molecular determinants are incompletely understood (1, 26, 27). Deciphering the localization and mobility of Vps10p-D receptors inside a cell and directly observing their interactions at spatial and temporal levels to examine local dynamics by live imaging has been challenging. We observed that C-terminal tagging of SorLA results in limited colocalization with the known interacting proteins PICK1 or GGA2. In contrast, the internally tagged SorLA construct colocalized with PICK1 and GGA2, respectively. A free C terminus is a prerequisite for binding of several PDZ-domain proteins, such as PICK1, to their C-terminal interaction motifs (65), and the interactions of GGAs with cytoplasmic domains depend also on a free C terminus (64). Therefore, the reduced colocalization between SorLA with a C-terminal GFP tag and PICK1 or GGA2 is probably because of a failure to interact. It is evident that interfering with the interaction of adaptor proteins guiding the intracellular itinerary of sorting receptors likely results in altered intracellular transport of the sorting receptors and thus altered functionalities. Hence, studies using C-terminal tags should be reevaluated.

We followed the strategy to tag the Vps10p-D receptors within the extracellular moiety. To maintain function and ligand-binding properties of tagged receptors, the correct folding of their individual domains is essential. Protein folding, along with different protein modifications, occurs cotranslationally and post-translationally in the ER by several chaperoning proteins, and the functional protein is then sorted from the ER to the Golgi apparatus (76). Misfolding of proteins generally prevents their exit from the ER, leads to accumulation in the ER, and eventually to a stress response and degradation. We predicted the structures of mVenus- and tdTomato-tagged Vps10p-D receptors computationally and observed their exit from the ER suggesting correct domain folding of the tagged receptors. Only the SorCS3 construct was partially trapped in the ER when expressed in COS7 cells or HeLa cells but not in HEK293 cells and primary cultured neurons. SorCS3 is the only Vps10p-D family member with an exclusive expression in the brain (6), and it is tempting to speculate that in some non-neuronal cells, such as COS7 cells, specific factors for successful folding of the receptor are lacking.

We specified the intracellular localizations of the tagged receptors, and our observations are largely in agreement with previous studies assessing subcellular localization of wt or chimeric Vps10p-D receptors by immunocytochemistry. Notably, detailed subcellular localization analyses have not been performed for all Vps10p-D receptors so far. Immunocytochemical studies turned out difficult in particular for SorCS1 and SorCS3 because both proteins are highly homologous, and specific antibodies for each receptor are not available. The new internally tagged receptor constructs are suitable tools to follow both receptors independently. We show significant colocalization of all fluorophore-tagged Vps10p-D receptors, but not of SorLA, with the CGN marker protein GM130. Still, the absence of SorLA colocalization with GM130 corroborates previous investigations (77). In agreement with previous studies, we observed that the receptors are targeted from the Golgi apparatus to the plasma membrane and to various endosomes of which several were Rab5 and Vps35 positive (6, 34, 45, 47, 51, 54, 69, 77, 78, 79, 80). Only SorCS1b, which is lacking internalization motifs in its cytoplasmic tail, is predominantly localized to the plasma membrane (33). Specific post-Golgi and postendocytic pathways are so far not completely characterized for all Vps10p-D receptors. The itineraries of Sortilin and SorLA are best characterized. Both interact directly with the retromer complex (45, 51). This is likely reflected in the high colocalization of fluorophore-tagged Sortilin and SorLA with the retromer subunit Vps35. Fluorophore-tagged SorCS1c-α, SorCS2, and SorCS3 demonstrated a significantly lower colocalization, presumably because of an indirect interaction with the retromer complex, indicated by the lack of canonical retromer interaction motifs in their cytoplasmic domains. However, co-IP of Vps35 with SorCS1 has been observed (81), supporting SorCS1 transport in Vps35-positive endosomes.

In accordance with a number of other studies, we found predominant somatodendritic expression of all fluorophore-tagged Vps10p-D receptors in primary cultured neurons (6, 77, 82, 83, 84, 85). However, we also observed additional expression of SorCS1b and SorCS3 in the axonal initial segment, which is in accordance with a previous investigation for SorCS1b (85).

The receptors of the SorCS subgroup homodimerize and heterodimerize through their leucine-rich domains (32). The demonstration of the homodimerization of fluorophore-tagged SorCS2 suggests functional leucine-rich domains and hints at an equally functional dimerization of fluorophore-tagged SorCS1 or SorCS3. Our successful FLIM application, also demonstrating interaction with BDNF, suggests the use of fluorophore-tagged Vps10p-D receptors in further interaction studies based on FRET–FLIM experiments in fixed and living cells.

The internal insertion sites established here can also be used for other tags, including application-tailored fluorophores with special photophysical properties. Thus, live cell analysis can be expanded by the introduction of alternative fluorophore tags, such as pH-sensitive fluorophores. These could be used to follow the receptors in late endosome and endolysosome trafficking and in postendocytic processes. Moreover, the genetically encoded tags can be introduced into the genome of the receptors by gene editing to avoid overexpression effects such as mislocalization to additional subcellular compartments.

The live imaging experiments performed here demonstrated the applicability of the designed fluorophore-tagged Vps10p-D receptors in live assays. For all tagged receptors except for SorCS1b, our data show *in vivo* cotransport with GFP-Rab5 as well as internalization of GFP-tagged BDNF. These observations corroborate the endocytic function of all Vps10p-D receptors, but not SorCS1b, and their transport through Rab5-positive early endosomes. We observed an endocytic uptake of biotin-labeled mature BDNF by all fluorophore-tagged Vps10p-D receptors but not SorCS1b, which promotes accumulation of mature BDNF on the cell surface. In the executed experiments, we focused on the functionality of the Vps10p-Ds in the tagged receptors. Accordingly, saturating concentrations of mature BDNF–biotin were used in the internalization experiments, and binding of lower affinity ligands might already result in receptor internalization. In addition, cells expressing either fluorophore-tagged Vps10p-D receptors, except SorCS1b, internalized BDNF-GFP secreted by cocultured cells. The BDNF-GFP construct encoded full-length BDNF, and internalized BDNF-GFP might correspond to both pro and mature BDNF-GFP. We could not detect TrkB in the employed HeLa and HEK293 cells by immunoblotting. This and the lack of internalization of BDNF in SorCS1b-transfected cells suggest that internalization of BDNF through the tagged Vps10p-D receptors was TrkB independent. However, additional studies involving, for example, TrkB knockout cells are needed to validate this assumption. Moreover, the FLIM experiments strongly suggest a direct interaction of all Vps10p-D receptors with BDNF. An interaction of SorLA and SorCS1 with a neurotrophin has not been demonstrated before, and we validated the direct binding of mature BDNF to the purified ectodomain of SorCS1. Taken together, our analyses show that BDNF is a shared ligand of all Vps10p-D receptors. All five Vps10p-D receptors mediate BDNF uptake and could at least act as scavenger receptors, but additional functional implications, as already demonstrated for Sortilin and SorCS2, need to be considered. Future systematic studies should determine the respective binding affinities of other neurotrophins and their proforms to each Vps10p-D receptor and investigate their potential role in neurotrophin signaling.

In summary, we designed functional fluorophore-tagged Vps10p-D receptors. We demonstrate that placing a tag at the internal sites used preserves several functionalities of the receptors, including the predicted folding and ER exit, subcellular localization, receptor dimerization and ligand interaction, and uptake and transport. The insertion sites are probably also suitable for other tags, and the functionalities of the constructs make them suitable as blueprints for gene editing approaches and may be applied in other experimental models, such as primary cells, induced pluripotent stem cells, or organoids. Using these internal tagging sites avoids several limitations of terminal tags. However, the newly developed constructs may still bear functional restrictions in so far unknown mechanisms. Overall, the internally fluorophore-tagged Vps10p-D receptors are novel powerful tools for future accurate surveys of the individual roles of the receptors in intracellular sorting and interaction with other proteins, which might be key to understand cytopathological mechanisms underlying neuronal and metabolic diseases.

## Experimental procedures

### Constructs for protein expression and structure prediction

To generate expression constructs, tdTomato or mVenus, as well as one N-terminal and one C-terminal fragment of (human) hSortilin, hSorLA, hSorCS1b, hSorCS1c-α, (murine) mSorCS2, and hSorCS3, respective complementary DNAs (cDNAs) were amplified by PCR using Q5 High-Fidelity DNA Polymerase (New England Biolabs). Using appropriate primers generating an ApaI site at the 3′-ends of cDNA encoding the N-terminal fragments of the Vps10p-D receptors and at the 5′-ends of tdTomato or mVenus cDNA. To the 5′-ends of cDNA encoding the C-terminal fragments and to the 3′-ends of tdTomato or mVenus cDNA, an XbaI site was added. For hSortilin, hSorCS1b, hSorCS1c-α, mSorCS2, and hSorCS3, the N-terminal fragment contained their extracellular domains, and the C-terminal fragment comprised their transmembrane and cytosolic domain, respectively. The transition point to the respective transmembrane domain was predicted using the TMHMM 2.0 (86, 87) and the TopPred tool (71, 72). Accordingly, the transition from the extracellular domain to the transmembrane domain of hSortilin is at V^757^ (UniProtKB ID: Q99523-1), for the hSorCS1 splice variants at G^1100^ (UniprotKB ID: Q8WY21-1), for mSorCS2 at Y^1080^ (UniprotKB ID: Q9EPR5-1), and for hSorCS3 at S^1124^ (UniprotKB ID: Q9UPU3-1). However, to enhance correct protein folding, C-terminal fragments of hSorCS1 and hSorCS3 were extended until the next proline, which often functions as a structure breaker between domains (88). Hence, the C-terminal fragments of the hSorCS1 variants start from P^1096^ and of hSorCS3 from P^1114^. For hSorLA (UniprotKB ID: Q92673-1), the N-terminal fragment contains the propeptide and the Vps10p-D (M^1^–E^759^), and the C-terminal fragment contains all domains starting with the YWTD repeat to the C terminus (F^760^–A^2214^). cDNAs encoding the respective N-terminal and C-terminal fragments were first cloned into the pcDNA3.1/Zeo(−) vector (Invitrogen). Between these two fragments, the amplified cDNA of tdTomato or mVenus was inserted respectively. For lentiviral expression, these constructs were then also cloned into the L21 vector (50). We generated first a SorCS3-tagged construct starting the C-terminal fragment following the fluorophore with S^1123^, but this was retained in the ER in different cell types. SorLA with a C-terminal GFP tag (SorLA CT-GFP) was cloned by eliminating SorLA’s stop codon by PCR and cloning the SorLA cDNA without a stop codon into pEGFP-N1 (BD Bioscience Clontech). The subcellular markers were all tagged at their termini. Accordingly, cDNA encoding GFP was cloned to the 3′-ends of the respective cDNAs encoding BDNF (89), TrkB, Vps35, or β-1.4-GT and cloned into an L21C-M2 destination vector. GFP-Rab5 (90), tdTomato-GGA2, and tdTomato-PICK1 (50) have been described before. mCherry-Rab9a was purchased from Addgene (Plasmid #78592) (91). The ER marker, tdTomato-tagged calreticulin signal peptide carrying a KDEL sequence, was a generous gift from Jakob Gutzmann. The tdTomato-GluA2 construct encodes the TGF-beta signal peptide followed by tdTomato and the last transmembrane and cytosolic domain of GluA2. Respective fragments were PCR amplified and cloned into pcDNA3.1/Zeo(−).

The structures of the wt sorting receptors were predicted using the AlphaFold Structure Database. To confirm correct protein folding of the tagged proteins, their structures were predicted employing the Robetta server and AlphaFold 2.0 (70, 92, 93). Because of computational limitations, however, the tagged protein predictions contain only the critical neighboring domains of the inserted fluorophores, respectively.

### Cell culture and transfection

COS7 (DSMZ; ACC60), HeLa (DSMZ; ACC57), and HEK293 (DSMZ; ACC305) cells were cultured at 37 °C and 5% CO_2_ in Gibco Dulbecco's modified Eagle's medium (DMEM; Thermo Fisher Scientific) supplemented with 10% fetal calf serum (FCS; Capricorn Scientific GmbH) and 100 U ml^−1^ penicillin–streptomycin (Thermo Fisher Scientific). SH-SY5Y (DSMZ; ACC209) cells were cultured at 37 °C and 5% CO_2_ in DMEM supplemented with 20% FCS. Transfection was performed using Lipofectamine 2000 (Invitrogen) according to the manufacturer’s protocol.

For primary hippocampal cell culture, hippocampi were dissected from E16 embryos of pregnant C57BL/6J mice collected in 10 mM glucose, rinsed with Hanks’ balanced salt solution (HBSS; Thermo Fisher Scientific), and treated with 0.05% trypsin–EDTA (Thermo Fisher Scientific) for 5 min at 37 °C. Trypsin reaction was stopped using 10% FCS in HBSS, and neurons were dissociated in HBSS by triturating with fire polished Pasteur pipettes. HBSS was removed, and approximately 60,000 cells/cm^2^ were seeded in PNGM (Lonza) supplemented with 100 U/ml penicilin–streptomycin on poly-l-lysine coated 12 mm glass coverslips. After 3 days *in vitro*, neurons were incubated with PNGM (Lonza) supplemented with NSF-1 (Lonza) for 30 min and transfected with 3 μg plasmid DNA using 125 mM CaCl_2_ in HeBS (137 mM NaCl, 5 mM KCl, 0.7 mM Na_2_HPO_4_, 7.5 mM glucose, 21 mM Hepes [pH 7.12]) for 40 min per coverslip. Transfected neurons were then fixed and immunostained 7 to 14 days *in vitro*.

### Immunocytochemistry, antibodies, and conjugates

Cells were fixed using ice-cold 4% paraformaldehyde (PFA) and 4% sucrose in PBS or with ice-cold methanol for 20 min and permeabilized with 0.05% Triton X-100 in PBS for 30 min. For immunostaining, the following antibodies were used: rabbit α-dsRed (to stain tdTomato) (catalog no.: 632496; Clontech; 1:500 dilution); chicken α-GFP (to stain mVenus) (catalog no.: ab139701; Abcam; 1:5000 dilution); mouse α-GM130 (catalog no.: ab169276; Abcam; 1:100 dilution); rabbit α-Rab5 (catalog no.: ab18211; Abcam; 1:200 dilution); rabbit mouse α-LAMP1 (catalog no.: 555798; BD Biosciences; 1:2000 dilution); chicken α-MAP2 (catalog no.: ab5392; Abcam; 1:10,000 dilution); mouse α-Ankyrin G (catalog no.: sc-12719; Santa Cruz; 1:100 dilution); goat α-chicken Alexa Fluor 488 (catalog no.: A11039; Invitrogen; 1:400 dilution); goat α-rabbit Alexa Fluor 555 (catalog no.: A21428; Invitrogen; 1:400 dilution); goat α-mouse Alexa Fluor 555 (catalog no.: A21422; Invitrogen; 1:400 dilution); and goat α-mouse Alexa Fluor 633 (catalog no.: 35513; Thermo Scientific; 1:400 dilution).

### Immunoblotting

To verify qualitative expression of indicated proteins, transfected or nontransfected cells were harvested and lysed in lysis buffer (150 mM NaCl, PBS [pH 7.4], 2% NP-40, 0.1% SDS, 0.5% sodium deoxycholate, 1 mM PMSF, and cOmplete proteinase inhibitor cocktail [Roche]) for 1 h at 4 °C. Protein concentrations of lysates were determined using the bicinchoninic acid method (BCA Protein Assay; Pierce). Equal amounts of protein were mixed with SDS sample buffer, boiled at 95 °C for 5 min, separated in 8% acrylamide gels, and transferred on polyvinylidene fluoride membranes. Specific proteins were then detected with respective primary antibodies and horseradish peroxidase (HRP)–conjugated secondary antibodies and visualized by ECL with SuperSignal West Pico PLUS Chemiluminescent Substrate (Thermo Scientific) using a Fujifilm LAS 4000 mini. For detection, the following antibodies were used: α-dsRed (to detect tdTomato) (catalog no.: 632496; Clontech; 1:1000 dilution); chicken α-GFP (to detect mVenus) (catalog no.: ab139701; Abcam; 1:10,000 dilution); mouse α-TrkB (catalog no.: 610101; BD Biosciences; 1:1000 dilution); rabbit α-pTrkA(Y^674/675^)/TrkB(Y^706/707^) (C50F3) (catalog no.: 4621; Cell Signaling; 1:250 dilution); mouse α-β-actin (catalog no.: A5441; Sigma; 1:1000 dilution); goat α-rabbit HRP (catalog no.: 65-6120; Invitrogen; 1:5000 dilution); rabbit α-chicken HRP (catalog no.: W4011; Promega; 1:5000 dilution); and goat α-mouse HRP (catalog no.: W4021; Promega; 1:5000 dilution).

### co-IP

Transfected cells were harvested and lysed with co-IP lysis buffer (150 mM NaCl, PBS [pH 7.4], 0.5% Triton X-100, 1 mM PMSF, and cOmplete proteinase inhibitor cocktail). A fraction of the lysates was saved and supplemented with SDS sample buffer as the input fraction, and the remaining lysates were coupled to GFP-Trap Nanobodies/VHH coupled to magnetic agarose beads (ChromoTek) by inverting them for 30 min at 4 °C. A part of the supernatant was saved and supplemented with SDS sample buffer. The beads were washed five times with dilution buffer (150 mM NaCl, PBS [pH 7.4], 1 mM PMSF, and cOmplete proteinase inhibitor cocktail) and finally supplemented with SDS sample buffer as the precipitate fraction. For visualization, input and supernatant fractions as well as the precipitates were immunoblotted.

### FLIM microscopy

To assess dimerization, HeLa cells were transfected with mVenus-tagged SorCS2 and either tdTomato-tagged SorCS2 or Sortilin. To monitor BDNF binding, one set of HeLa cells was transfected with BDNF-GFP and another set of HeLa, HEK293, or SH-SY5Y cells transfected in parallel with the tdTomato-tagged Vps10p-D receptors or tdTomato-GluA2, respectively, and cocultivated 1 day post transfection. Two days post transfection, cells were fixed with 4% PFA in PBS for 20 min, confocal images were taken, and fluorescence lifetimes of the mVenus fluorophores were measured using an Abberior STED-confocal expert line system (Abberior Instruments). The images were acquired in confocal mode. Pixel by pixel, the fluorescence lifetime intensity curve was acquired. An image of the sample was obtained by summing up the fluorescence lifetime intensity curves associated to each pixel.

During the pixel-by-pixel analysis, an equal intensity threshold was set, to exclude from the analysis lifetime profiles that signal-to-noise ratio was too poor to provide reliable estimates. In the end, only the pixels above the threshold were analyzed. Statistical differences between lifetimes were validated through Kruskal–Wallis analysis with a post hoc Mann–Whitney *U* test using IBM SPSS Statistics 25. For lifetime images, pixels above the threshold were color coded based on their fluorescence lifetimes. Normalized frequencies were calculated as the relative number of pixels at a lifetime in a range of 0.01 ns in relation to the amount of pixels at the respective lifetime with the maximum amount of pixels.

### BDNF uptake assay

HeLa cells or HEK293 cells were transfected with tdTomato-tagged Vps10p-D receptors, respectively. Two days post transfection, cells were starved for 2 h in Opti-MEM without FCS (OptiMEM, Thermo Fisher Scientific). Subsequently, the cells were cooled down to 4 °C and incubated for 1 h with 0.1 μg/ml ice-cold hBDNF–biotin (B-250-B; Alomone Labs Ltd) in OptiMEM for receptor binding at the plasma membrane. Nonbinding hBDNF–biotin was removed by washing twice with ice-cold OptiMEM. For internalization of the receptors, the cells were incubated 10 min at 37 °C, followed by immediate fixation with 4% PFA and 4% sucrose in PBS at 4 °C. hBDNF–biotin was visualized by using streptavidin, DyLight 488 conjugated (catalog no.: 21832; Thermo Scientific; 1:500 dilution).

For live-cell analysis of BDNF uptake, one set of HeLa cells was transfected with BDNF-GFP and another set in parallel with the tdTomato-tagged Vps10p-D receptors, respectively. About 24 h post transfection, the two differently treated sets of cells were cocultured with a ratio of 40% BDNF-GFP-expressing cells and 60% tdTomato-tagged Vps10p-D receptor–expressing cells. After another 24 h, live time-lapse images were taken from HeLa cells expressing the tdTomato-tagged Vps10p-D receptors.

**Fig S3:**
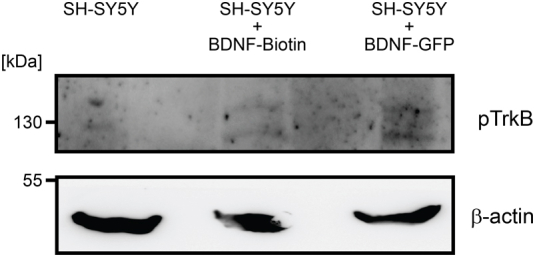
**BDNF-Biotin and BDNF-GFP elicit TrkB activation.** TrkB-expressing SH-SY5Y cells were cultured in serum-free media for 6 h, followed by treatment with control serum-free medium, BDNF-Biotin or medium containing BDNF-GFP for 2 h. Lysed SH-SY5Y cells were analyzed by immunoblotting using a phospho-TrkB–specific antibody (*upper panel*) and β-actin (*lower panel*).

Biological activity of BDNF–biotin and BDNF-GFP was assessed by TrkB activation. HeLa cells were transfected with BDNF-GFP, and 6 h post transfection, cells were washed and medium was replaced with serum-free DMEM (starvation medium). About 2 days post transfection, BDNF-GFP was harvested by collecting the starvation medium from the transfected HeLa cells, followed by centrifugation and filtering through a 0.2 μm PES filter to remove detached cells. SH-SY5Y cells were starved for 6 h with starvation medium. Then the medium was exchanged with either fresh starvation medium (control), starvation medium supplemented with 200 nM BDNF–biotin or, freshly harvested starvation medium containing BDNF-GFP. After 2 h, SH-SY5Y cells were washed twice with ice-cold Tris-buffered saline and lysed for 30 min at 4 °C with Tris lysis buffer (10 mM Tris [pH 7.5], 150 mM NaCl, 0.5 mM EDTA, 0.5 mM EGTA, 2% NP-40, 0.1% SDS, 0.5% sodium deoxycholate, 1 mM PMSF, cOmplete proteinase inhibitor cocktail, and phosphatase inhibitor PhosSTOP [Roche]). Protein concentrations of lysates were determined, and lysates were analyzed by immunoblotting using a phospho-TrkB–specific antibody (Fig. S3).

### Confocal imaging and colocalization

Immunofluorescent images were taken by confocal microscopy using a TCS SP8 microscope (Leica) with a 60× magnification objective. For time-lapse microscopy, cells were seeded in glass bottom dishes and transfected with indicated fluorophore-tagged fusion proteins. During live-cell imaging, temperature was kept at 37 °C and CO_2_ levels at 5%. Time-lapse movies were taken with the TCS SP8 microscope and formatted using Fiji ImageJ.

For colocalization analysis, laser power and gain were kept identical between images. Using the Fiji ImageJ plugin JaCoP, the Mander’s coefficients for colocalization were calculated. These represent the amount of immunoreactivity of one channel colocalizing with the immunoreactivity of the other channel. Bar graphs were produced using GraphPad Prism 5 (GraphPad Software, Inc). Statistical differences were validated through one-way ANOVA with post hoc Tukey tests for parametric datasets or Kruskal–Wallis and post hoc Mann–Whitney *U* tests for nonparametric datasets in IBM SPSS Statistics 25.

### Surface plasmon resonance analyses

The extracellular moiety of SorCS1 was expressed and purified as described before (33). Measurements were performed on a BIAcore 3000 instrument equipped with CM5 sensor chips as described (33, 94) with the purified extracellular moiety of SorCS1 immobilized to a density of approximately 50 fmol/mm^2^. BDNF (Alomone Labs Ltd) was injected at 5 μl/min in 10 mM Hepes, 150 mM NaCl, 1.5 mM CaCl_2_, 1 mM EGTA, 0.005% Tween-20, pH 7.4. The overall *K*_*d*_ (dissociation constant) was determined by BIAevaluation 3.0 software (BIAcore) using a Langmuir 1:1 binding model.

## Data availability

All data are contained within the article and are available upon reasonable request. The generated plasmids will be deposited at Addgene.

## Supporting information

This article contains supporting information.

## Conflict of interest

The authors declare that they have no conflicts of interest with the contents of this article.

## Supporting information

1_GFPRab5_Sortilin Confocal live image analysis of a COS7 cell transfected with Sortilin-tdTomato (*magenta*) and GFP-Rab5 (*green*) representing cotransport of Sortilin and Rab5. Scale bar represents 10 µm. Images were recorded with a framerate of 2.2 fps and are represented with a framerate of 2 fps.

2_GFPRab5_SorLA Confocal live image analysis of COS7 cells transfected with SorLA-tdTomato (*magenta*) and GFP-Rab5 (*green*) representing cotransport of SorLA and Rab5. Scale bar represents 10 µm. Images were recorded with a framerate of 2.16 fps and are represented with a framerate of 2 fps.

3_GFPRab5_SorCS1c-a Confocal live image analysis of a COS7 cell transfected with SorCS1c-α-tdTomato (*magenta*) and GFP-Rab5 (*green*) representing cotransport of SorCS1c-α and Rab5. Scale bar represents 10 µm. Images were recorded with a framerate of 4.97 fps and are represented with a framerate of 2 fps.

4_GFPRab5_SorCS2 Confocal live image analysis of COS7 cells transfected with SorCS2-tdTomato (*magenta*) and GFP-Rab5 (*green*) representing cotransport of SorCS2 and Rab5. Scale bar represents 10 µm. Images were recorded with a framerate of 4.48 fps and are represented with a framerate of 2 fps.

5_GFPRab5_SorCS3 Confocal live image analysis of a HEK293 cell transfected with SorCS3-tdTomato (*magenta*) and GFP-Rab5 (*green*) representing cotransport of SorCS3 and Rab5. Scale bar represents 10 µm. Images were recorded with a framerate of 2.16 fps and are represented with a framerate of 2 fps.

6_GFPRab5_SorCS1b Confocal live image analysis of a COS7 cell transfected with SorCS1b-tdTomato (*magenta*) and GFP-Rab5 (*green*) representing no cotransport of SorCS1b and Rab5. Scale bar represents 10 µm. Images were recorded with a framerate of 4.39 fps and are represented with a framerate of 2 fps.

7_BDNFGFP_Sortilin Confocal live image analysis of a HeLa cell transfected with Sortilin-tdTomato (*magenta*) cocultured with HeLa cells transfected with BDNF-GFP (*green*) representing vesicular cotransport of Sortilin and BDNF. Scale bar represents 10 µm. Images were recorded with a framerate of 1.71 fps and are represented with a framerate of 2 fps.

8_BDNFGFP_SorLA Confocal live image analysis of a HeLa cell transfected with SorLA-tdTomato (*magenta*) cocultured with HeLa cells transfected with BDNF-GFP (*green*) representing vesicular cotransport of SorLA and BDNF. Scale bar represents 10 µm. Images were recorded with a framerate of 1.72 fps and are represented with a framerate of 2 fps.

9_BDNFGFP_SorCS1ca Confocal live image analysis of a HeLa cell transfected with SorCS1c-α-tdTomato (*magenta*) cocultured with HeLa cells transfected with BDNF-GFP (*green*) representing vesicular cotransport of SorCS1c-α and BDNF. Scale bar represents 10 µm. Images were recorded with a framerate of 1.73 fps and are represented with a framerate of 2 fps.

10_BDNFGFP_SorCS2 Confocal live image analysis of a HeLa cell transfected with SorCS2-tdTomato (*magenta*) cocultured with HeLa cells transfected with BDNF-GFP (*green*) representing vesicular cotransport of SorCS2 and BDNF. Scale bar represents 10 µm. Images were recorded with a framerate of 1.71 fps and are represented with a framerate of 2 fps.

11_BDNFGFP_SorCS1b Confocal live image analysis of a HeLa cell transfected with SorCS1b-tdTomato (*magenta*) cocultured with HeLa cells transfected with BDNF-GFP (*green*) representing colocalization of SorCS1b and BDNF on the cell surface. Scale bar represents 10 µm. Images were recorded with a framerate of 1.71 fps and are represented with a framerate of 2 fps.
